# Core formation resolves Earth’s siderophile excess without a late veneer

**DOI:** 10.1126/sciadv.aef0411

**Published:** 2026-09-25

**Authors:** Ingrid Blanchard, Julien Siebert, Lucas Calvo, Sergey S. Lobanov, Coline Pinchon, Edith Kubik, Max Wilke, Anja Schreiber, Valentina Bonino, François Guyot, Sylvain Petitgirard, Paraskevas Parisiades, Nicolas Guignot

**Affiliations:** ^1^ Sorbonne Université, Muséum National d’Histoire Naturelle, UMR CNRS 7590, Institut de Minéralogie, de Physique des Matériaux et de Cosmochimie (IMPMC), Paris, France.; ^2^ Institut für Geowissenschaften, Universität Potsdam, Potsdam, Germany.; ^3^ Institut de Physique du Globe de Paris, Université Paris Cité, Paris, France.; ^4^ GFZ Helmholtz Centre for Geosciences, Potsdam, Germany.; ^5^ Bayerisches Geoinstitut, Universität Bayreuth, Bayreuth, Germany.; ^6^ UCD School of Earth Sciences, University College Dublin, Dublin 4, Ireland.; ^7^ ESRF-The European Synchrotron, Grenoble, France.; ^8^ Muséum National d’Histoire Naturelle, Sorbonne Université, UMR CNRS 7590, Institut de Minéralogie, de Physique des Matériaux et de Cosmochimie (IMPMC), Paris, France.; ^9^ Department of Earth Sciences, ETH Zürich, Zürich 8025, Switzerland.; ^10^ Synchrotron SOLEIL, Gif-sur-Yvette, France.

## Abstract

Highly siderophile elements (HSEs) are expected to be extensively depleted in the mantle due to their affinity for metal during core formation. Yet, their abundances in the mantle far exceed the predictions based on low pressure and low temperature partitioning experiments. In addition, all HSEs are present in similar chondritic relative proportion in the mantle. This has traditionally been attributed to the late accretion of chondritic material, the late veneer. We present laser-heated diamond anvil cell experiments that quantify the partitioning behavior of five HSEs (rhenium, osmium, iridium, palladium, and gold) under pressure-temperature conditions directly relevant to early Earth’s magma ocean. Our results demonstrate a substantial decrease in metal-silicate partition coefficients with increasing temperature. Multistage accretion models reproduce the observed HSE abundances and ratios in the mantle without invoking a late veneer. This resolves the longstanding paradox of HSEs excess’ in the mantle and suggests that the delivery of volatile compounds to the young Earth is primarily explained by early accretion and differentiation processes.

## INTRODUCTION

The highly siderophile elements (HSEs), including gold (Au), iridium (Ir), osmium (Os), palladium (Pd), rhenium (Re), rhodium (Rh), ruthenium (Ru), and platinum (Pt), are characterized by their strong affinity for metallic iron and their tendency to partition into metal-rich phases. Experimental studies at relatively low pressures and temperatures have consistently shown that HSEs exhibit very low solubility in silicate melts (*1*–*4*), with liquid metal–liquid silicate partition coefficients (*D* values, see eq. S1) exceeding 10^4^. Such *D* values suggest that during the primary differentiation of rocky planets, HSEs were largely sequestered into the metallic cores, leaving the silicate mantles comparatively depleted. In Earth’s case, more than 99.99% of its total HSE budget is estimated to reside in the core. Yet, the natural abundances of HSEs in Earth’s mantle are several orders of magnitude higher than predicted by equilibrium partitioning models alone, and the relative proportions of HSEs in the mantle closely match those found in chondritic meteorites (*5*, *6*). This apparent contradiction, commonly referred to as the excess siderophile element problem, poses a longstanding challenge in geochemistry, raising fundamental questions about the origin and timing of HSEs delivery to Earth’s mantle.

The dominant hypothesis to explain the mantle’s budget is that HSEs were added to Earth’s mantle after core formation ceased, through the accretion of chondritic material, commonly called the late veneer. This addition took place after the Moon-forming impact, contributing to a considerable mass supply of approximately 0.5 weight % (wt %) of Earth’s total mass (*6*–*8*). The late veneer would effectively replenish the mantle in HSEs, accounting for both their elevated abundances and the near-chondritic relative ratios observed today. In addition to explaining the HSE budget, this model also provides a plausible mechanism for the delivery of volatile elements, such as water and carbon, to the Earth, critical components for establishing planetary habitability (*9*, *10*). Supporting evidence includes chondritic Re/Os and Pt/Os isotopic ratios in mantle-derived rocks, consistent with a late veneer composed of undifferentiated chondritic material, after core formation (*11*).

There is no doubt that a certain amount of chondritic material has been supplied to the Earth’s surface after the differentiation of the core, since this influx continues to the present day. However, determining the mass of this late addition is linked to the leftover material remaining after differentiation of the core, and therefore to the not yet fully known partition coefficients of HSEs between metal and silicates. Although high-pressure experiments consistently yield large partition coefficients for HSEs, these studies are often conducted under conditions, such as moderate pressures (<20 GPa) and temperatures (<2500 K), along with elevated oxygen fugacities, that do not accurately represent the much higher pressure, temperature regime of early Earth differentiation (>50 GPa and >3500 K) (*12*–*14*). Consequently, extrapolating these results may introduce substantial errors. Theoretical predictions suggest that HSE partitioning behavior may converge and decrease substantially at the elevated temperatures typical of a deep magma ocean (*15*). To date, this has been convincingly demonstrated only for Pt, which shows decreasing siderophile behavior with increasing temperature (*16*). Whether similar temperature-dependent trends hold for other HSEs remains a critical question.

In this study, we address this gap by experimentally determining the metal-silicate partitioning of Re, Ir, Au, Pd, and Os under pressure-temperature conditions directly relevant to core formation. Using laser-heated diamond anvil cell (LH-DAC) techniques to simulate magma ocean depths, and silicate compositions representing the primitive mantle in equilibrium with multielement Fe-rich alloys, we measured the partitioning behaviors of these HSEs with state-of-the-art analytical methods. Our results reveal a strong temperature dependence in the partitioning for all investigated elements, with decreasing siderophile behavior at high temperatures. When integrated into geochemical models, these findings demonstrate that the present-day mantle abundances and near-chondritic relative ratios of HSEs can be accounted for by core formation alone, substantially reducing the required mass of a late veneer and thus calling into question its ability to supply important parts of the Earth’s water and carbon budgets.

## RESULTS

**Fig. 1.**
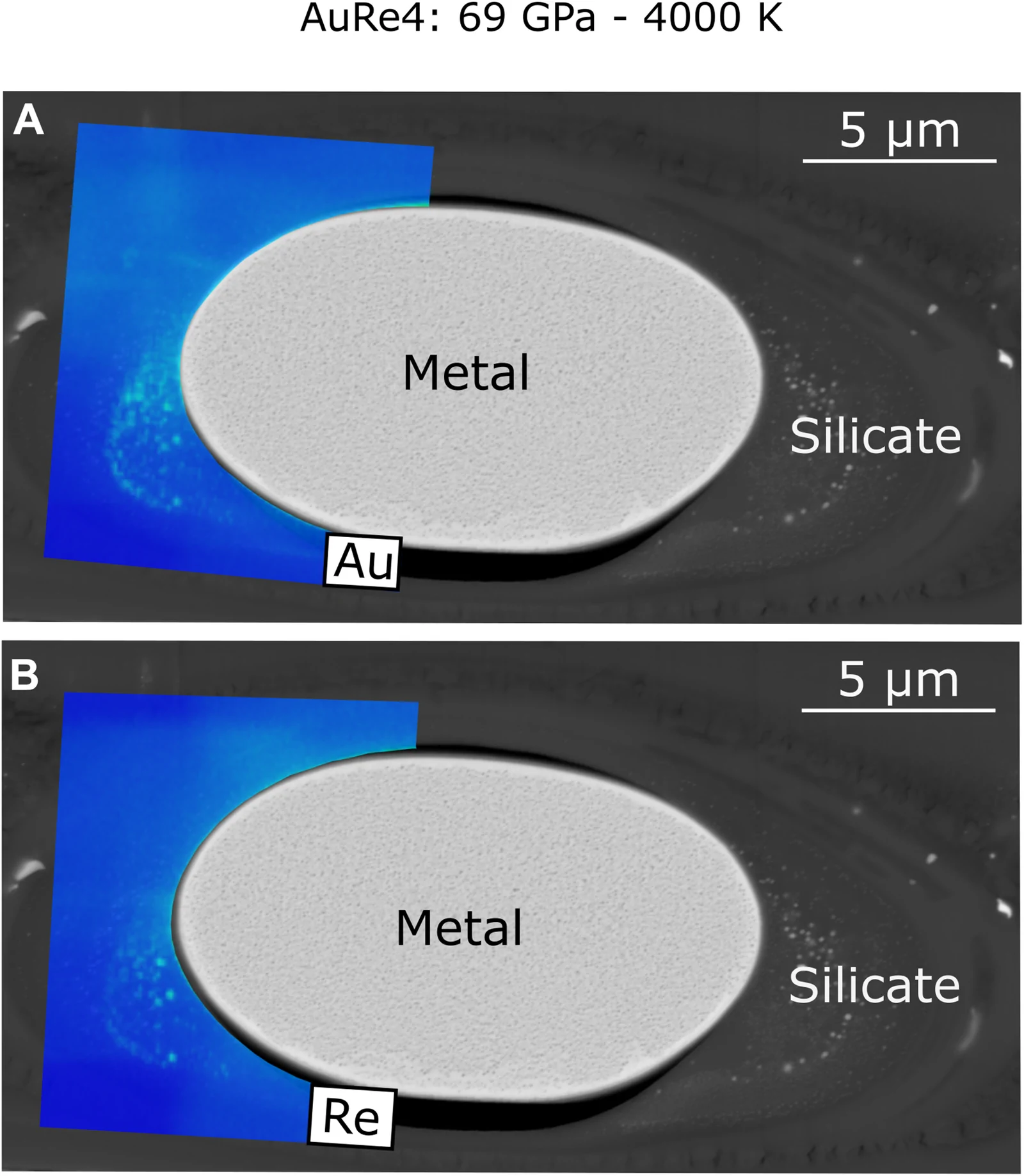
Recovered run and super imposed nano-XRF map. Sample AuRe4 (synthesized at 69 GPa and 4000 K) displays a central metallic phase surrounded by a silicate melt. We report nano-XRF maps for Au and Re on images (**A**) and (**B**), respectively.

**Fig. 2.**
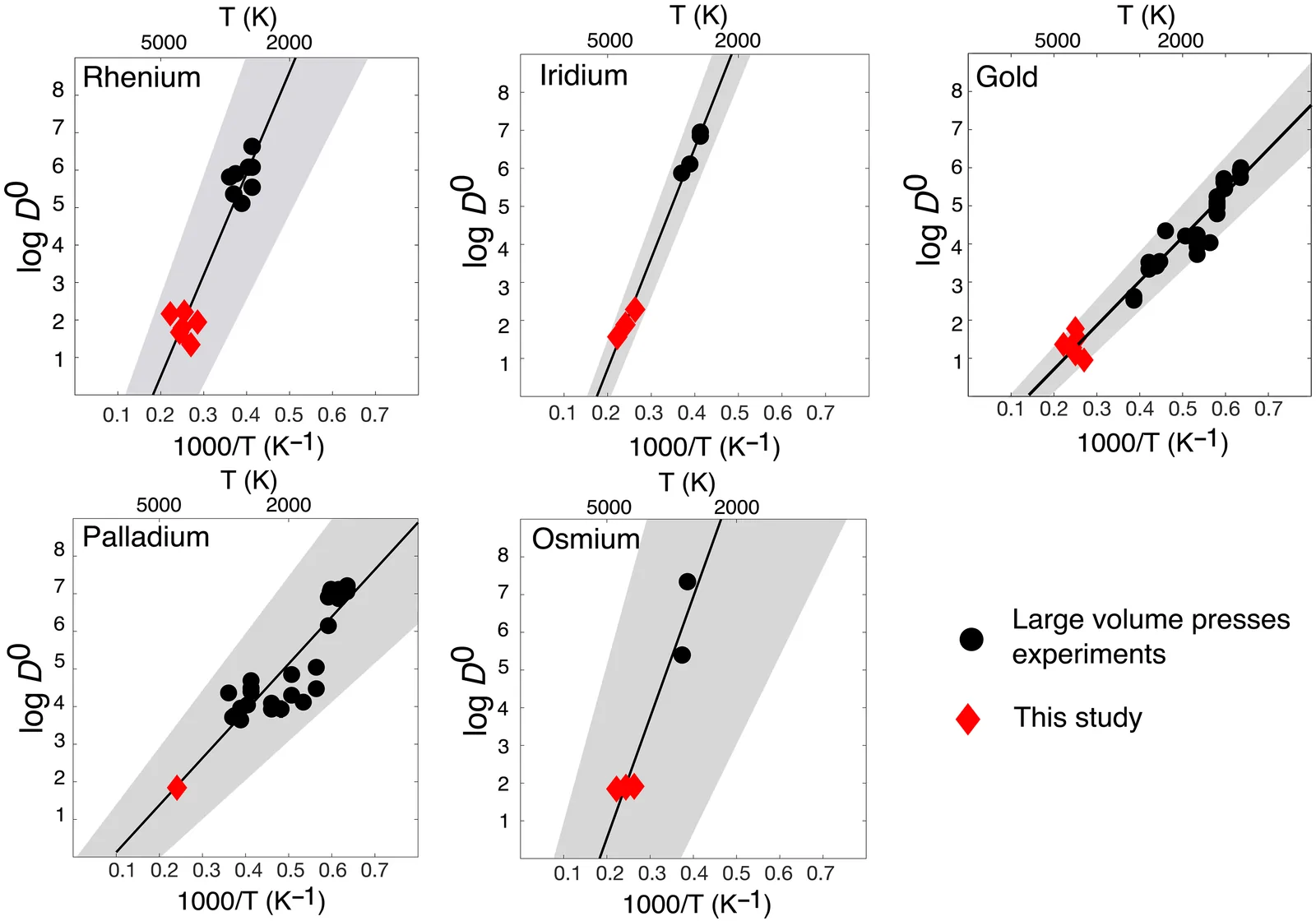
Evolution of *D*^0^ as a function of the inverse temperature for Re, Ir, Au, Pd and Os. Each panel represent in red the data obtained in this study, and in black data from the literature (*1*, *2*, *18*–*20*, *39*, *40*, *58*, *86*–*88*). Uncertainties are smaller than symbol size when not displayed.

We conducted 13 experiments using LH-DAC at pressures and temperatures ranging between 40 to 81 GPa and 3500 to 4500 K respectively (see the Supplementary Materials). Core-mantle equilibration under these extreme conditions has previously been shown to reproduce the observed mantle depletions of several moderately siderophile elements, including Ni, Co, W, and Mo (*12*, *17*). In addition, our experiments were conducted under reducing conditions similar to those expected during core formation (∼IW − 2, i.e., two log units below the iron-wüstite oxygen fugacity buffer, see the Supplementary Materials), in contrast to most previous studies, which were performed under more oxidizing conditions (above IW − 1) (*18*–*20*). Run products were recovered using a focused ion beam (FIB) and typically consisted of a spherical metallic blob (up to ∼15 μm in diameter) surrounded by a quenched silicate melt, ranging from ∼20 to 40 μm across (Fig. 1). This geometry is characteristic of superliquidus partitioning experiments, where both metal and silicate phases were fully molten and equilibrated at high temperature. Major element compositions of both phases were determined by energy-dispersive x-ray (EDX) analysis (EDS), while trace concentrations of HSEs in the silicate were quantified using nano–x-ray fluorescence (nano-XRF) at the upgraded ID16b beamline of the European Synchrotron Radiation Facility (ESRF). This beamline offers the spatial (<50 nm) and analytical [parts per million (ppm)–level] resolutions required to accurately measure trace HSE concentrations in samples of this scale (see tables S5 and the Supplementary Materials). Submicrometer metallic particles (<100 nm) dispersed in some localized regions of the silicate melt were observed in scanning electron imaging (SEM) and further characterized by transmission electron microscopy (TEM) coupled to EDS, which revealed that these nanoparticles exhibit Fe/HSE ratios approximately 40 times higher than that of the central metal blob (see the Supplementary Materials), indicating they are neither derived from nor in equilibrium with the main metal phase. We infer that these particles were initially dissolved in the silicate at superliquidus conditions and exsolved during rapid quenching. Similar quench features have also been reported and discussed in previous metal-silicate LH-DAC experiments (*21*), suggesting that they are not unique to HSE-bearing systems. Using measurements of HSE in both central metal blob and silicate areas, partition coefficients for all five HSEs were determined in each experimental run and are reported in table S3, using $D_{M}=\frac{X_{M}^{\text{metal}}}{X_{M}^{\text{silicate}}}$, where *X*_*M*_ is the molar fraction of the element in the respective phase. To correct for compositional effects and reflect HSE behavior at infinite dilution, more representative of natural core-forming systems, we applied a Margules mixing model for binary or ternary alloys (*18*, *22*), as described in the supplementary materials. The obtained recalculated partition coefficients, *D*^0^, represent values appropriate for core-forming conditions and are also reported in table S3. Under the conditions of our experiments, and systematically for all HSEs studied, the partition coefficients are lower by several orders of magnitude compared to previous results obtained at lower P-T conditions (Fig. 2). Our experimental results display *D* values that can already account for the excesses of these elements in the bulk silicate earth (BSE; see table S3). Some of our experiments contain several HSEs, others are limited to one HSE; nevertheless, all experiments converge toward similar partition coefficients. Our data sets for all HSEs plot on a single linear trend, respectively, which is a strong function of temperature (Fig. 2), while pressure has a much smaller effect. The presence of oxygen and silicon dissolved in the metallic phase of our runs does not substantially alter this behavior. We report in the methods the temperature dependence for all five HSEs.

## DISCUSSION

Our experimental results were incorporated into multistage core formation models to investigate the behavior of HSEs during Earth’s accretion. In these models, Earth grows through a series of impacts with progressively larger planetary embryos, consistent with the dynamical evolution of the protoplanetary disc (*23*). The impactor masses range from 0.1 to 10% of Earth’s mass, reflecting the increasing size of colliding bodies as accretion proceeds. More details on the model are given in the Supplementary Materials. Our model produces mantle concentrations that are coherent with estimation for the BSE. We calculate final values after core formation of 0.350 ± 0.005, 2.40 ± 0.05, 1.800 ± 0.005, 8.03 ± 0.07, 11.00 ± 0.04 parts per billion of Re, Os, Ir, Pd, and Au, respectively, very close to the ones of the BSE (*24*, *25*) (0.26 ± 0.07, 3.9 ± 1.2, 3.6 ± 1.0, 4.8 ± 0.7, and 1.7 ± 0.5).

**Fig. 3.**
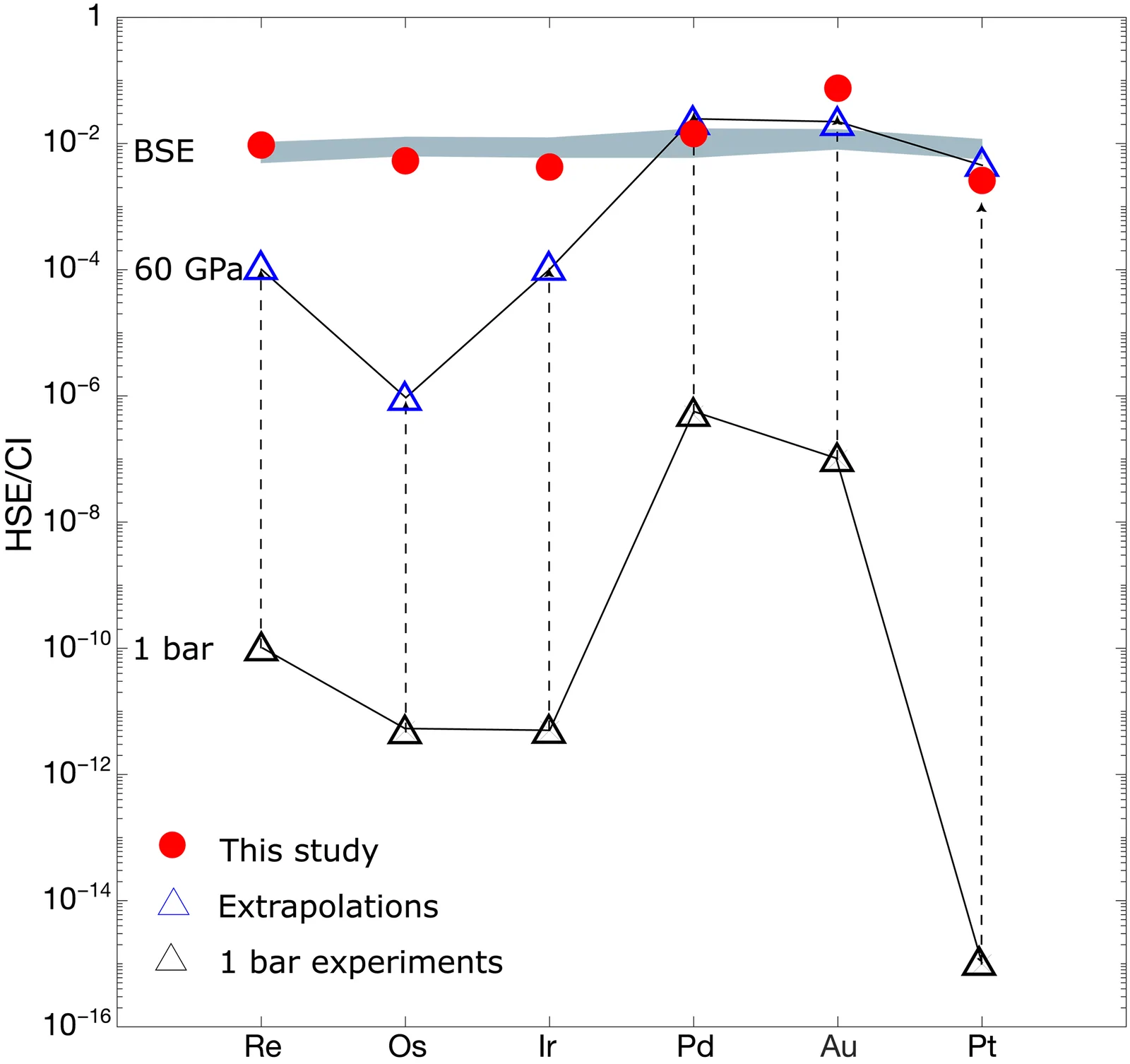
Comparison of HSE/CI ratios from this study with estimates from the literature. Outputs from our accretion model (red dots) display a chondritic relative abundance comparable to the BSE ratios (*24*, *25*). For comparison, previous 1 bar experiments (*1*–*3*, *26*–*28*) and extrapolations (*18*–*20*) are shown.

Figure 3 shows the modeled concentrations of each HSE in the BSE at the end of our core formation model, normalized to CI chondrite abundances. For comparison, we also plot estimated HSE concentrations in the BSE retrieved from geochemical compilations (*24*, *25*), as well as predictions based on extrapolations from lower P-T partitioning experiments (*18*–*20*) and melting experiments conducted at 1 bar (*1*–*3*, *26*–*28*). The model results show remarkable agreement between our calculated HSE concentrations and the BSE estimates. It also reproduces the chondritic relative pattern, which was not the case for previous extrapolations (*18*–*20*). We also incorporated high-pressure data on Pt, obtained under conditions directly relevant to core formation (*16*). Together, these results demonstrate that the mantle abundances of Re, Os, Ir, Pd, Au, and Pt can be accounted for by metal-silicate equilibration during accretion. Our study shows that core formation alone can reproduce the observed mantle inventory of HSEs, indicating that a late chondritic veneer is not required to explain their present-day abundances.

A key argument in support of the late veneer hypothesis is its ability to explain the chondritic relative abundances of HSEs in the Earth’s mantle. In particular, Os isotope systematics constrain the relative abundances of Re and Os in the BSE. The measured ^187^Os/^188^Os and ^186^Os/^188^Os ratios lie within the range of chondritic meteorites, indicating that Re and Os must have very similar metal-silicate partition coefficients, within approximately 5% for Re and Os and 40% for Pt and Os. While our experiments do not achieve the precision required to confirm this level of similarity in *D* values for Re and Os, they provide direct evidence that chondritic Re/Os and Pt/Os ratios can result from core-mantle equilibration alone. Our results show that metal-silicate equilibrium during core formation can reproduce both the isotopic and elemental ratios observed in the mantle, with final values of Re/Os = 0.14 ± 0.03 and Pt/Os = 1 ± 0.3. While not exactly similar to the mantle estimates [0.090 ± 0.002 for Re/Os (*24*) and 1.9 ± 0.57 for Pt/Os (*11*)] these values are very close, which is a major improvement compared to previous estimation based on lower P-T experiments who predicted ratios orders of magnitude different (*20*).

The evolution of HSE abundances in Archean komatiites has been cited as evidence for the late veneer, yet when examined in detail, the collected data present a more complex picture. While a general increase in HSE concentrations over time is observed [e.g., (*29*–*32*)], several late Archean komatiites, such as those from Schapenburg (∼2.9 Ga) and Kostomuksha (∼2.7 Ga), exhibit lower HSE contents than some early Archean komatiites, including those from the Isua supracrustal belt (∼3.7 Ga) and Barberton greenstone belt (∼3.5 to 3.2 Ga), and are inconsistent with the amount of late veneer inferred from Os and W isotopes (*30*, *33*). Similarly, although komatiites from Pilbara and Barberton have been interpreted to reflect a mantle retaining a pre-late veneer signature, the relatively high HSE abundances in Isua samples challenge the idea of a globally unmixed mantle reservoir. Rather than recording a temporal trend, the observed variations in HSE abundances may instead reflect sampling of mantle sources at different depths that retained early chemical signatures established during magma ocean crystallization and core formation (*34*). Recent Ru isotope data suggest that Earth’s core and its current mantle show distinctive composition (*35*), with Earth’s mantle evolving from its early composition due to the addition of a carbonaceous chondrite-like late veneer (*35*). This requires a modest late veneer addition of no more than ∼0.2 wt % of Earth’s mass (*36*), which is consistent with our model, as developed below. However, the lack of partitioning data for Ru at relevant conditions limits the reliability of these estimates. Recent calculations based on geophysical and astrophysical arguments have demonstrated the contradiction between the observed concentration of HSEs in the mantle and the previous estimation of 0.5 wt % of accreted late veneer (*37*). Overall, these observations support our conclusion that a large-scale late veneer is not necessary to explain Earth’s mantle composition in HSEs.

Our results do not rule out the possibility of a minor late veneer component. To evaluate the amounts of late veneer compatible with the residual mantle abundances after core formation, we performed a statistical analysis (see the Supplementary Materials). This analysis shows that up to ∼0.2 wt % of Earth’s mass could have been added after core formation without conflicting with geochemical constraints, and would be sufficient to explain the HSE inventory of the Earth. Nevertheless, Au concentrations in the mantle are already exceeded at the end of our model. This is similar to earlier studies based on lower pressure and temperature experiments (*38*–*40*), and calls for mechanisms to remove them from the mantle. To do so, two processes have been suggested that may have lowered Au concentrations in the BSE, as a deep magma ocean crystallizes. On the one hand, sulfide segregation during Earth’s early differentiation, known as the “Hadean Matte” (*41*, *42*). This mechanism relies on the high temperature and sulfur-dependent behavior of HSEs, as their partitioning is influenced not only by temperature but also by the sulfur content of the metal phase (*43*). However, for sulfide segregation to occur, the magma ocean must have reached sulfide saturation. Recent high-pressure, high-temperature experiments suggest that sulfide saturation was unlikely to have been achieved during Earth’s accretion (*44*, *45*). This challenges the viability of the Hadean Matte model and suggests that it cannot account for the observed HSE abundances in Earth’s mantle. On the other hand, iron disproportionation (*46*) has been proposed to generate enough metallic iron to deplete the BSE in HSE (*16*). Yet, recent experiments performed at conditions relevant for Earth’s core formation have shown a less efficient self-oxidation effect at deep magma ocean conditions (*47*).

A simpler explanation for Au anomaly may reside in our poor knowledge of its abundance in BSE, since it can be the most incompatible of the HSEs and is also more susceptible to modification via melt depletion and percolation. In addition, none of the chondrite groups provides a good match to BSE estimates (*24*, *48*).

Late accretion appears to be a common feature in the evolution of differentiated bodies across the Solar System, with evidence for late-stage impacts found on the Moon, Mars, and asteroids such as Vesta. The Moon notably offers a unique record of a potential late veneer, as its core formed under much lower pressure–temperature conditions than Earth’s, where HSEs remained highly siderophile and were efficiently scavenged from the mantle during core formation. Lunar mantle-derived rocks show extreme depletion in HSEs compared to Earth’s mantle, by at least a factor of 20 [e.g., (*49*)], with estimates as low as ∼0.0002× CI chondrite (*50*). These values are substantially below those expected if the Moon had accreted a proportion of late material scaled by gravitational cross section. Some models attribute the low HSE content to delivery by a few large impactors, which would preferentially accrete to Earth and strip material from the Moon (*51*, *52*). Dynamical simulations have proposed that reproducing the Earth-Moon HSE ratio requires one or a few very large impactors (diameter >1000 km). Yet, such large, differentiated bodies would have merged their HSE-rich metallic cores with Earth’s core and likely triggered large-scale mantle melting. This is inconsistent with multiple geochemical observations, including the survival of ancient zircons (older than 4.38 Ga) and isotopic evidence for early crustal differentiation by ∼4.45 Ga (*53*, *54*) ago. Moreover, models suggesting that the Moon-forming impactor that delivered HSEs to the Earth’s mantle requires its core to have remained unmixed with Earth’s, which contradicts impact simulations and experimental results (*55*, *56*). Martian mantle HSE abundances, estimated from SNC meteorites, are lower than those of the Earth by roughly a factor of two (*57*), and can be explained by alternative mechanisms, including a single giant impact or the formation of a sulfur-rich core under reducing conditions (*58*, *59*). Similarly, HSE signatures in HED meteorites from Vesta exhibit a wide variability, with some showing signs of contamination, but no consistent evidence for a late veneer (*11*, *60*, *61*). While late accretion certainly occurred to varying extents across the inner Solar System, the low-mass late veneer inferred for the Moon and possibly other terrestrial bodies aligns well with our results, which highlight a dominant role for core formation in generating the Earth’s mantle HSE inventory, while still allowing for a minor late addition.

The timing of volatile element delivery to Earth has been a central topic of debate over recent decades. One view holds that volatiles were delivered gradually during the main stages of accretion (*62*, *63*), while another proposes that they arrived primarily during a late veneer phase (*9*, *10*). However, this latter scenario has been increasingly challenged by both modeling and experimental evidence. Recent studies show that the BSE abundances of volatile elements such as sulfur, carbon, and chalcogens can be explained by processes active during core formation and early differentiation (e.g., (*21*, *64*, *65*)). Our findings support this view by showing that the mantle HSE budget can be explained without invoking a substantial late veneer. By extension, this suggests that volatile delivery to Earth, including critical elements for habitability like hydrogen, carbon, and nitrogen, occurred primarily during the earlier phases of planetary accretion and core formation. This implies that the presence of key life-supporting elements on Earth is not necessarily dependent on the final stages of planetary growth, but rather on earlier stages of accretion and differentiation.

## MATERIALS AND METHODS

### Starting materials

We have synthetized our silicate and metal starting materials using levitation furnace device and piston cylinder respectively. We describe in the following the two procedures.

The silicate starting material was a synthetic glass with either the composition of a mid-ocean ridge basalt (MORB), or of a pyrolite (P1 and P3) synthetized by a levitation furnace device in Orléans, France (*66*). We report in table S1 their composition, measured by electron microprobe analysis at the University of Bayreuth. We have subsequently polished down these glasses to a thickness of 20 μm and machined them at Institut de Physique du Globe de Paris, France (IPGP), to obtain small disks of 80 to 100 μm diameter (*67*).

As for the starting metals, four different alloys were synthesized using piston-cylinder. The first mixture (MET1) was 60 wt % Fe + 30 wt % Re (PURATRONIC, 99.999 %) and 10 wt % Au (Premion, 99.998 %). The second one (MET2) was made with 80 wt % Fe + 20 wt % Os (99.8%, Thermo Fisher Scientific), the third (MET3) was made from 80 wt % Fe + 20 wt % Ir (99.99%, VWR), and the last one (MET4) was made with 60% Fe + 10% Pd (>99.995 purity, VMR) and 30% Ir (99.99% VWR).

Those four combinations were subsequently mixed separately with a synthetic MORB, melt and equilibrated in a piston-cylinder in IPGP, to help the coalescence of a central metallic sphere, and facilitate the extraction of the synthesized alloys. Proportions were 40% of MORB mixed with 60% of Fe-HSEs mixtures. For Au, Re and Pd-bearing starting material, powders were melted at 2 GPa and 1873 K in an MgO capsules for 10 min using a ½″ piston-cylinder apparatus at IPGP. For Ir- and Os-bearing starting material, samples were kept at 2 GPa and 2073 K for 5 min in MgO capsule, also at IPGP. Their composition was checked by EPMA (table S1) at the University of Potsdam for MET1 and MET4 and by EDS at IPGP for MET2 and MET3. We used pure Re, Au, Os, Pd, Ir, and Fe as standards, and counted 20 s on the peak and 10 s on the background for each element. As can be seen on fig. S1, all metals developed a micrometer-size texture upon quenching. This explains the rather high SD observed for MET1. For MET2 and MET3, we used raster of about 10 μm to integrate the whole composition. These results also in lower SD reported in table S2.

The quenched alloys were then crushed and used as the metallic portion of ours experiments. The inhomogeneity of our starting metals is reflected in the various concentration of HSEs in the recovered alloy part of our LH-DAC experiments. We report on table S3 which starting material combination was used for each experiment.

### LH-DAC experiments

We performed 13 successful LH-DAC experiments between 42 and 81 GPa and 3500 to 4500 K. We used diamonds with 200- to 300-μm culets and rhenium gaskets. The gaskets were pre-indented to obtain a thickness of 40 to 50 μm, and were subsequently laser-drilled to produce a sample chamber ∼90 to 120 μm in diameter. We loaded two silicate disks sandwiching a flake of one of the HSE-rich alloys into this chamber (*21*, *67*). Samples were compressed to the target pressure and then laser-heated at the desired temperature using a doubled-sided laser-heating system either at GFZ Helmholtz Centre for Geosciences (Germany), Institut de Minéralogie et de Physique des Matériaux et de Cosmochimie (France, IMPMC), at the PSICHE beamline of the synchrotron SOLEIL (France), or IPGP (France).

The use of four experimental setups for laser heating using different lasers, focusing optics, and detectors allowed us to verify that the measured partitioning coefficients are mostly sensitive to temperature, and are largely independent of other experimental parameters (e.g., laser spot size, numerical aperture of the focusing optics, sample temperature characterization). In the following, we give more information regarding the different laser heating systems.

At the GFZ, we used a 1070-nm IPG photonics fiber laser in a bench-top setup (*68*) that features an optical Pi-shaper to deliver a nearly flat-top laser profile across ∼20 to 30 μm. The thermal emission was spatially filtered to the area under the flat-top laser, collected and recorded by a Teledyne Princeton Instruments HRS-300 spectrometer with a PIMAX-4 detector recording 100-μs frames at 5 Hz [first frame was collected ∼50 ms after the onset of laser heating, sufficient for thermal equilibration (*69*)]. Fitting the 600 to 900 nm spectral data of the individual frames to Planck function under the graybody approximation yielded temperatures and their typical variations of 200 to 400 K over the heating cycle, which we took into account when estimating the overall temperature uncertainty of 300 K. The use of the graybody approximation is appropriate because the available constraints on the emissivity of silicate melts at high pressure-temperature conditions (albeit scarce) suggest the emissivity is nearly wavelength-independent in the spectral range of our temperature measurements (*68*).

At IMPMC, the samples were heated simultaneously from both sides by two continuous YAG fiber lasers (wavelength 1064 μm), providing a maximum combined power of 200 W.

The experimental procedure has been devised to minimize the temperature gradients in the samples. The laser spot size was slightly defocused to 20 μm diameter to obtain a more uniform heating of the sample and reduce the temperature gradients due to the Gaussian shape of the TEM00 mode of the lasers. Therefore, the reported temperatures should be understood as spectroradiometric temperatures averaged over the heated region, rather than exact point temperatures. The alignment of laser and pyrometry spot were verified at every heating cycle and the temperature between the two sides was kept very similar by tuning λ/2 wave plates for each side as we gradually increased the laser power. The spectral range of the fit for the collected Planck radiation was between 600 and 800 nm. At the IMPMC, we used reflective Schwarzschild optics, without any chromatic aberration. The spectral response of the optical system was calibrated prior to the experiments by using a tungsten-halogen lamp.

At the PSICHE beamline, temperature measurements were performed using the four-color pyrometry system available on PSICHE [as also described in (*70*)]. This setup enables real-time acquisitions of temperature and emissivity maps at the sample surface, which were used to control radial temperature gradients. Chromatic aberrations are kept minimal thanks to the use of reflective objectives. Laser beamshapers were used to generate a flat-top profile at the focal point. Typical hot spot diameters were in the range of 15 to 30 μm.

At IPGP, we used a 200-W infrared fiber laser, that was split it two to heat from both sides simultaneously. Temperatures were continuously measured by spectroradiometric technique (*71*) The spectral response of the optical system was calibrated before experiments by using a tungsten-halogen lamp.

Pressure was determined using the Raman shift of the diamonds (*72*), which allowed us to avoid using a ruby chip in the experiments. At all facilities, temperature was measured simultaneously and continuously on both sides of the diamond cell, using a spectro-radiometric technique (*71*). Temperature was increased above the liquidus temperature of the sample silicate [pyrolite or basalt (*73*, *74*)], held for a few tens of seconds and then the lasers were switched off to promote quenching. This short equilibration time is enough, given the small size of our runs (*67*). The samples quenched extremely quickly due to the high thermal conductivity of the diamond anvils, and were subsequently decompressed slowly over several hours. We estimated that the temperature uncertainty was ±300 K (*75*). The estimate of the final pressure was corrected for thermal pressure following ∆*P* = 2.7 MPa/K (*73*, *74*).

### Sample recovery

We used the Focused Ion Beam facility (FIB, DualBeam Helios G4 UC Thermo Fisher Scientific/FEI) at GFZ to recover small lamellae of 3 μm thickness, about 40 μm in length and 20 μm wide, from the samples using a Ga^+^ ion beam (*76*). In the samples, we observed distinct quenched metal and silicate phases that had both been molten and equilibrated at high P-T (see Fig. 1A in the main text). The sections were then studied by SEM and synchrotron-based XRF at the nanoscale (nano-XRF). For checking with higher spatial resolution, we investigated selected samples by TEM.

At this step, we were able to see the sample for the first time, which allowed us to clearly understand if the experiment was successful or not. We need the samples to display a quenched metallic phase and a quenched silicate phase, both of them big enough (several micrometers) to be further analyzed by SEM-EDS. In addition, extracted samples had to display a parallel geometry on a 3-μm-thick lamella, to have reliable measurements with EDS. In total, we produced about 30 samples, but only 13 of them were successful.

### Field-emission gun measurements

Major elements in both the metallic and the silicate phase were measured using EDX analysis with a Zeiss Cross-beam Field Emission Gun at IPGP. The operating conditions were 15 keV, and samples were coated with a thin layer of carbon before analysis. Both silicate and metal phases are big enough to obtain reliable EDX measurements. The FIB lamellae are thick (∼3 μm), which also ensures consistent EDX measurements (*77*). Several spectra were taken on each phase for 60 s and quantified using standards. The average composition of the metal and the silicate phase of all our runs is reported on table S4. We observed that samples are homogeneous at the scale of analysis, which is an indication that chemical equilibration was closely approached. As observed previously, the molten silicate displays an enrichment in FeO with respect to the starting material, which results in redox conditions of −0.67 to −1.46 relative to the IW buffer (*16*, *17*, *21*, *67*, *78*), see more discussion below. The metal phase is composed mostly of iron, alloyed with HSEs. As can be seen in table S4, the concentration of HSEs in the metallic phase is highly variable, ranging from about 1 wt % to more than 45 wt %. To adjust for these high concentrations, we have corrected our runs using a Margules based formalism (see the section “Thermodynamics” in the Supplementary Materials). As can be seen in table S4, recovered experiments conducted with the same starting material display variable amounts of HSEs in the metallic phase. This is a direct consequence of the heterogeneous nature of all the starting metal, which is visible on fig. S1 and table S2.

Also present in the metallic phase are oxygen (∼3.5 to 6.5 wt %) and silicon (0 to ∼7 wt %).

### TEM measurements

Our main goal with these analyses was to image and analyze the dispersed metallic droplets present in the silicate melt, often referred to as “nano-nuggets” (*79*). This is an important aspect of our understanding of metal-silicate partitioning of HSEs elements. Several works already focused on the interpretation of HSEs nuggets in silicate melts at lower P-T conditions, and so far, no consensus was achieved (*26*, *80*–*82*). The fundamental question is to understand whether those nuggets are formed upon quenching, or if they are the result of saturation. In the first case, they should be integrated into the composition of the silicate phase and used to assess the amount of HSE dissolved in it, whereas in the second case they should be avoided during the silicate analysis.

We note that this kind of nuggets has been observed in many previous LH-DAC experiments from the literature where metal and silicate melts are equilibrated without the presence of HSEs (*12*, *17*, *21*). Hence, in the case of LH-DAC experiments, those nuggets are more related to the technic itself than to the nature of the element considered. This being said, it is essential to assess the composition of those nuggets, especially in this study where we investigate the metal-silicate partitioning of HSEs.

To better understand the origin of nuggets observable in our runs (as in Fig. 1), we have used TEM analysis. To do so, we further thinned down sample FeIr1 to a 100-nm-thick lamella using FIB. The TEM observations were carried with a Jeol 2100 F transmission electron microscope operating at 200 kV, equipped with a field emission gun, a high-resolution UHR pole piece, a Gatan US4000 CCD camera, and an EDX analysis system from JEOL, which allowed us to perform electron diffraction and imaging as well as elemental maps in STEM mode.

As previously reported (*21*), the quenched silicates display a bimodal distribution of metallic nuggets. The two populations are very different in various ways. First, they have very different sizes, and second, they have very different compositions.

The first ones are relatively large particles 100 to several 100 nm across irregularly dispersed in the silicate (circled in purple in fig. S2B), whereas the second ones are much smaller droplets (few tens of nm across) uniformly dispersed in the quenched silicate melt (circled in green in fig. S2B). We have undertaken to measure both populations to compare their respective compositions in HSEs. EDS analyses of the large inclusion highlight a composition close to the central metallic sphere. We interpret this feature as being present at high temperature during the experiments, as entrained metal. Conversely, analyses of the small droplets dispersed in the silicate are very different, with a Fe/Ir ratio more than 40 times higher than the central metallic sphere. Those are interpreted as quench features, representative of the silicate composition. Hence, during our nano-XRF analyses (see next paragraph), we have taken particular care in avoiding the big inclusions, but have incorporated the smaller ones into the composition of the silicate melts.

We stress that this interpretation is in line with several previous studies containing HSE such as Pt (*16*), or without HSEs (*21*, *83*).

### Nano-XRF measurements

To measure the concentration of HSEs in the recovered silicate phase of our runs, we carried out nano-XRF measurements at the beamline ID16B, at ESRF in Grenoble, France. We used a highly focused pink beam (Δ*E*/*E* = 10^−2^) and high brilliance at an excitation energy of 29.5 keV. This configuration makes this technique particularly suitable for analyses of heavy elements such as HSEs (*76*). The nanosized beam (about 60 × 60 nm) was scanned through each sample, and the XRF was collected by two multielemental Si drift detectors. High-resolution maps, as the one shown in Fig. 1, were collected. Several measurements campaigns at the ESRF synchrotron were performed. Two detectors are used, and they are positioned at 10° from the sample’s surface. Each time, we used the central element present on each detector, to maximize the signal, and avoid shadowing effects. As discussed in the former paragraph, during both the nanoXRF and field-emission gun analyses, we took particular care in avoiding large metallic nuggets that are sometimes dispersed in the quenched silicate. For each sample, we selected multiple regions of interest (ROIs) that were devoid of any large metallic nuggets. Concentrations of HSEs for each experiment is a mean of all those measurements. Before proceeding with data treatment, we corrected our data for detector deadtime using PyMca software developed by ESRF (*84*). This software was also used to extract summed XRF spectra for ROIs on each sample. Since samples were also analyzed for major elements, we used the internal calibration in PyMca to determine the concentration of HSEs in silicate phases. This procedure was verified by measurements on FIB-lamellae of reference glasses [see below, and (*76*)].

We present on fig. S3 a typical spectrum obtained by PyMca software on an Au and Re-rich sample (ESRF, equilibrated at 49 GPa and 4000 K). We highlighted the L lines of Au and Re that allowed us to determine their concentration in the silicate phase. As seen on fig. S3, the XRF spectra is well fitted, and we can clearly resolve individual peaks of Au and Re.

To check our ability to measure HSEs in our recovered runs, we have used a reference sample with known composition in HSEs (*85*). We extracted a 3-μm-thick FIB-lamellae out of it. We present in table S5 the values that are expected versus what we measure using nano-XRF. For Ru, Rh, Ag, and Re, values agree within 10 to 15%. For Pd and Ir, values deviate by ca. 50%. Osmium is present in only small amount (6 ppm), which seems too low to be analyzable by this method as the measured value deviates by a factor of more than 4. The elements for which the reproducibility is the best are the ones that are present at the 100-ppm level or more. This is very encouraging, since in our experimental runs, we always measured 1000-ppm level of HSEs in the silicate phase.

Estimation of the detection limit for each sample is not straightforward. The presence of a large metallic bleb in the center of the sample that is HSE-rich can also result in fluorescence that might pollute the silicate signal of HSE. We have tested this by measuring in our experimental run the abundance of the HSE of interest in the unreacted part of each sample. This zone should be HSE-free, since it did not react with the metallic part. We have always found values of about 200 to 400 ppm. This indicates that the central HSE-rich metal contributes to the signal, but there is always about an order of magnitude difference between measurements in the molten silicate and the unreacted region. Hence, we believe that our measurements are robust, and the best that can be achieved with this technique.

### Valence state

Estimation of valence state for HSEs in these experiments is not straightforward, and should rely on multiple experimental data obtained at similar P-T conditions, over a wide range of redox conditions. From our samples, we cannot determine the valence state of each element, so we relied on previous estimations. Valence state decreases with decreasing fO_2_ (*18*), and at high redox state, it was proposed that HSEs can have high valence state, such as 4+ for Re at IW + 3 (*26*). Here, we have redox conditions much closer to the one prevailing during core formation, with fO_2_ around IW-1 for all our experiments. Following previous estimation made for Pt at similar P-T conditions (*16*), we have assumed that all our HSEs were at a zero-valence state at our experimental conditions.

## Supplementary Materials

### This PDF file includes:

Supplementary Text Figs. S1 to S7 Tables S1 to S7 References
