# Supplementary material for: Core formation resolves Earth’s siderophile excess without a late veneer
Source: Sci Adv. 2026 Sep 25;12(39):eaef0411. doi: 10.1126/sciadv.aef0411 (PMC13614371; doi:10.1126/sciadv.aef0411)
Supplement: Supplementary file 1 [file sciadv.aef0411_sm.pdf]

Science Advances
AAAS

Supplementary Materials for

Core formation resolves Earth's siderophile excess without a late veneer

Ingrid Blanchard *et al.*

Corresponding author: Ingrid Blanchard, ingrid.blanchard@sorbonne-universite.fr

Sci. Adv. **12**, eaef0411 (2026)
DOI: 10.1126/sciadv.aef0411

This PDF file includes:

Supplementary Text
Figs. S1 to S7
Tables S1 to S7
References

Supplementary Text

Thermodynamics

The thermodynamic treatment that we used follows the one described elsewhere (*18*). We explain in the following the main steps, but more details can be found in the aforementioned publication. The partitioning of an element M between metal and silicate in our experimental runs is represented by its molar partition coefficient D_{exp}, which can be written:

$D_{exp} = \frac{X_M^{metal}}{X_{MOn/2}^{silicate}}$ (Eq. S1),

with X the molar fraction of element M in the metal or in the silicate.

To make sure we could measure the concentration of HSEs in both the metal and the silicate phase, we had to start with high concentration of HSEs in the metallic starting material (see table S2). These concentrations are much higher than what is expected for natural system and require consideration of thermodynamic activity composition relations. Indeed, since the elements of interest are not present at trace concentration level of infinite dilution, the activity coefficient of HSEs deviates from Henryan behavior, and changes as a function of X_M^{metal}. Hence, each experimental partition coefficient D_{exp} needs to be recalculated into values corresponding to infinite dilution. In order to do so, the metal activity coefficient at the level of infinite dilution ($\gamma_M^{0,metal}$) and the activity coefficient (γ_M^{metal}) in the Fe-rich alloys need to be determined for each experiment. Subsequently, one can calculate D^0, the metal-silicate partition coefficient corresponding to HSE metal concentration at the level of infinite dilution which is representative of core-mantle differentiation conditions. D^0 is expressed as:

$D^0 = D_{exp} \cdot \frac{\gamma_M^{metal}}{\gamma_M^{0,metal}}$ (Eq. S2).

We have estimated γ_M^{metal} and $\gamma_M^{0,metal}$ using Margules-type mixing model (*18*, *22*). This model is an attempt to best model Fe and HSEs interactions, and we had to make several simplifications. To our knowledge, it is the best way to recalculate our experimental D values to infinite dilution. We have done the following simplifications (*18*, *40*): since there are no thermodynamic data for any liquid binary involving Re, we have used available data on Ru instead. Indeed, solid solutions with Fe involve a hcp phase close to the liquidus for both Re and Ru, so we assumed that the mixing behavior with Fe in the liquid state is very similar for both elements (*18*). For Au, we assumed that Au behaves as Pd (*58*), and used values reported in (*27*) from the primary data of (*89*). For Pd and Ir, we have used Margules parameters presented in (*18*). Iron-Osmium thermodynamic data are extremely rare; hence we directly used the excess energy of mixing (*90*).
We have used the ternary formalism for experiments where two HSEs were present simultaneously, and the binary formalism for experiments where only one HSE was present.

The excess energy of mixing G_m^{xs} of any solution at a pressure P (in bar) and temperature T (in K) is generally defined as:

$G_m^{xs} = \Delta H_m - T\,\Delta S_m^{xs} + (P-1)\Delta V_m^{xs}$ (Eq. S3)

With ΔH_m the enthalpy of mixing, ΔS_m^{xs} the excess entropy of mixing and ΔV_m^{xs} the excess volume of mixing. Following (*22*), G_m^{xs} of a binary and ternary system composed of elements M, N (and O) can also be described with an asymmetric regular solution model following the two respective equations:

$G_m^{xs} = X_M X_N (X_M W_{NM} + X_N W_{MN})$ (Eq. S4)

$G_m^{xs} = X_M X_N (X_M W_{NM} + X_N W_{MN}) + X_M X_O (X_M W_{OM} + X_O W_{MO}) + X_O X_N (X_O W_{NO} + X_N W_{ON}) + X_M X_N X_o C_{MNO}$ (Eq. S5)

Where X_M and X_N, X_O are the mole fractions of the components, and Ws are asymmetric Margules parameters (see table S4). CMNO is defined as:

$C_{MNO} = \frac{1}{2}(W_{MN} + W_{NM} + W_{MO} + W_{OM} + W_{NO} + W_{ON}) - W_{MNO}$ (Eq. S6)

Following (*18*), we have neglected the ternary Margules parameter W_{MNO}.
Those binary Margules parameters mainly depend on the enthalpy of mixing, but they also vary slightly with P and T, and can be expressed:

$W = W^{H,1bar} - T\, W^S + (P-1)W^V$ (Eq. S7)

With $W^{H,1bar}$, W^S and W^V referring to components of W related to the enthalpy of mixing at 1 bar, excess entropy of mixing and excess volume of mixing of the solution respectively. We have neglected W^V as they found these values to be very small (*18*).
Since most thermodynamic from the literature were collected at lower temperature (hereafter T^0) compared to ours we have extrapolated the values such as (*18*, *91*):

$W = (W^{H,1bar} - T^0\, W^S + (P-1)W^V)\frac{T^0}{T}$ (Eq. S8)

We have based our mixing model on equations 15 and 21 of ref. (*22*) applied to two or three components, in order to calculate γ_M^{metal}, such as:

$RT \ln \gamma_M = 2(X_M X_N W_{NM}) + X_N^2 W_{MN} - 2G_m^{xs}$ (Eq. S9)

for binary systems, and

$RT \ln \gamma_M = 2(X_M X_N W_{NM} + X_M X_O W_{OM}) + X_N^2 W_{MN} + X_O^2 W_{MO} + X_N X_O C_{MNO} - 2G_m^{xs}$ (Eq. S10)

for ternary systems.
The same mixing model is used to calculate $\gamma_M^{0,metal}$, but for conditions of X_{Fe}=1 and X_{HSE}=0. The values of the Margules parameters used in this study are presented in table S6.
Once γ_M^{metal} and $\gamma_M^{0,metal}$ are calculated, it is possible to assess the value of D^0 for each experiment, following equation S2.
In adequation with (*16*), we express D^0 as a function of temperature such as:

$\log D^0 = a + \frac{b}{T}$ (Eq. S11)

We present in fig. S4 a comparison of D and D^0, and highlight that the differences are always quite small.

Oxygen fugacity was calculated relative to the iron-wüstite buffer (IW) for each run following:

$\Delta IW = 2 \log \frac{X_{FeO}}{X_{Fe}}$ (Eq. S12)

with X_{FeO} and X_{Fe} the molar fraction of FeO in the silicate and Fe in the metallic phase respectively. As described previously, we have been careful in selecting zones that did not contain big metallic nugget, to avoid underestimating the fO_2.We have assumed ideal mixing of both phases, which is justified by the high temperatures of our runs. As displayed in Table S3, our experimental runs were equilibrated between IW-0.67 and IW-1.61. This range of fO_2 is very similar to previous metal-silicate partitioning studies (*12*, *17*, *67*), which is a strong argument to validate our calculation.

We have also evaluated whether our experiments reached, or closely approached, chemical equilibrium. To do so, we have calculated the equilibrium constant for silicon of our runs (K_{Si}), and compared them to previously published K_{Si}. We express K_{Si} such as:

$$K_{Si} = \frac{D_{Si}}{{D_{Fe}}^{2}}$$

K_{Si} is known to be strongly temperature dependent (*12*, *92*). We have used the MetalAct website (https://norrisau.org/expet/metalact/?p=about#WoodWade2005 (*13*)) in order to compute activity coefficients for Si in iron alloys.
We plot on fig. S5 our calculated K_{Si} for both our experiments and previous data from the literature spanning a wide range of temperature (2023-5000K). This figure highlights the perfect agreement of our data with previously published K_{Si}. We believe that this is the strongest argument we can make to prove that we closely approached equilibrium during our experiments.

Data selection

We compiled data from the literature obtained with large volume press experiments in order to encompass a wide range of pression and temperature conditions. We have used data that were obtained using MgO capsules, contained no sulfur in the metallic phase, and were synthetized at redox conditions similar to ours (between IW and IW-2).
We compiled data for gold (*2, 19, 20, 39, 40, 88, 93*), rhenium (*18*) , osmium (*19*, *87*), iridium (*18*), and palladium(*1, 2, 18, 58, 86*). Those data and ours were recalculated to infinite dilution following the procedure described previously, when it was not already proposed in the aforementioned papers.
Following the compilation all our dataset with the selected data, we obtain:

$$\log D^0_{Au} = -1.6\ (0.4) + \frac{11\,553\ (880)}{T} \quad \text{(Eq. S12)}$$

$$\log D^0_{Re} = -4.9\ (1.5) + \frac{27\,062\ (4\,458)}{T} \quad \text{(Eq. S13)}$$

$$\log D^0_{Ir} = -5.1\ (0.5) + \frac{29\,092\ (1\,611)}{T} \quad \text{(Eq. S14)}$$

$$\log D^0_{Os} = -6.0\ (2.7) + \frac{32\,006\ (9\,025)}{T} \quad \text{(Eq. S15)}$$

$$\log D^0_{Pd} = -1.1\ (1) + \frac{12\,523\ (2\,340)}{T} \quad \text{(Eq. S16)}$$

As can be seen in the equations S12 to S16, we do not observe any effect of the presence of light elements on the partitioning of HSEs. Despite the dissolution in the metallic phases of our run of several wt.% of O and/or Si, we could not extract a contribution of those elements to the metal-silicate partitioning of HSEs. Hence, the only parameter that influences the partitioning of those elements is temperature. This is coherent with results obtained on Pt at the same conditions (*16*).

Core formation model

Our new parameterization for Re, Au, Ir, Os and Pd metal-silicate partitioning was subsequently used in multi-stage core formation models. In those models, the Earth grows from the accretion of small bodies (planetesimals and planetary embryos). The proto-Earth mantle is melting due to intense impact heating and release of gravitational energy due to the formation

of the core itself. P-T conditions of core-mantle equilibration is increasing as the planet grows, along the peridotite solidus-liquidus.
Here, accretion is discretized in a large number of steps. For the first 80%, small increments of 0.1% of Earth's mass are added, followed by 2% increments, and a final 10% mass increment (the Moon-forming impact). This scenario is based on dynamical models (*94*), and has been used a vast number of studies (*17*, *95*, *96*).
Here, we want to stress that the core-mantle partitioning of HSEs is temperature dependent, and not mass dependent. The other end-member scenario is the pebble accretion scenario, that also happens at high temperatures (*97*). Hence, we are confident that our results are not strongly model-dependent.
The last pressure of equilibration at each step i, $P_{(i)}$, was calculated according to the final core–mantle boundary pressure, P_{CMB} (Eq. S17).

$$P_{(i)} = P_{CMB} f_{(i)}^{2/3} f_p \text{ (Eq. S17)}$$

Where $f_{(i)}$ is the accumulative mass fraction on Earth, and $f_p$represents the core–mantle proportion on Earth, which is 0.41. P_{CMB} is 135 GPa. For the temperature of equilibration, we have used the arithmetic average of the peridotite liquidus proposed by (*73*, *74*). Over the course of accretion, we assumed a constant fO_2 of IW-2.3. Following recent fluid dynamics estimation (*98*, *99*), we have considered the embryos and the proto-Earth to fully equilibrate as long as they are below 800 km in radius. For the last giant impact, no equilibration at all is assumed, and the core of the impactor merges directly with the one of the Earth. The effect of having no equilibration versus a little equilibration for the last giant impact is assessed on fig.S6. We present the evolution of log D for Re and Au as a function of the accreted fraction. As can be seen, there is little difference between a scenario where there is no equilibration assumed (k=0 for the last impact) or little equilibration (k=0.3 for the last impact).
Errors on the model are calculated by a Monte Carlo-like approach, where at each step of the accretion model log D^0 is calculated by picking random *a* and *b* values within their errors (Eq. S12 to Eq.S16) over 10 000 iterations.
Throughout the accretion model, we performed mass balance calculations to track HSEs abundance in Earth's core and mantle, with the assumption that accreting bodies had HSEs abundances equivalent to those of CI chondrites(*100*, *101*). In the following, concentration of each element j in the mantle and core (C_j^M and C_j^C, respectively) are calculated considering Earth's core and mantle mass (M) and fractions ($f^C = 0.323$, and $f^M = (1 - f^C)$, respectively) (Eq. S18).

$$C_j^{BE} M_j^{BE} = C_j^{BE} M_j^{BE} f^C + C_j^{BE} M_j^{BE} f^M \text{ (Eq. S18)}$$

Where BE stands for bulk Earth. Throughout the stepwise accretion model, the mass conservation of the accreted material was calculated for the mantle (Eq. S19) and the core (Eq. S20).

$$\frac{d}{dt}\left[(1 - f^C) M^{BE} C_j^M\right] = \left[(1 - f^C) C_j^{M-IMP} + \epsilon_j f^C \left(C_j^{C-IMP} - D_j^0 C_j^M\right)\right] \frac{dM}{dt} \qquad \text{(Eq. S19)}$$

$$\frac{d}{dt}\left[f^C M^{BE} C_j^C\right] = \left[(1 - \epsilon_j) f^C C_j^{C-IMP} + \epsilon_j f^C D_j^0 C_j^M\right] \frac{dM}{dt} \qquad \text{(Eq. S20)}$$

With IMP referring to the accreted impactors. ϵ_j is an equilibration parameter being 1 at each step, except for the giant impactor where is 0 as no equilibration is assumed. Eq S19 and S20

are calculated at each step and adds up to the accumulated concentration. Uncertainties on the abundances rise from the Monte Carlo-like approach mentioned previously.

Abundances of HSEs in Earth's mantle

Estimation of HSEs abundances in Earth's mantle is not straightforward. Different estimation methods can lead to variations of several tens of percent. Rock types investigated range from orogenic peridotite to cratonic xenoliths. Here, we have used the compilation of data (*34*). For each HSE, we have estimated the mean, the minimum and maximum abundance (*24, 102-106*).

Statistical analysis

Given the relatively large uncertainties associated to estimates for the Earth's mantle composition and with geochemical mass balance models, we evaluate the possibility of a certain mass addition after core formation in a late veneer fashion. Namely, we have statistically evaluated whether CI-like material added after our model matches estimates of HSEs abundance in the BSE.

We considered 0.1 wt.% mass increments from 0 wt.% to 0.5 wt.% with an additional intermediate step of 0.25 wt.%. On the one hand, we performed Student's t-tests to determine if the addition of each increment produced a mantle statistically different from the BSE. On the other hand, we performed Bayesian inference tests which yield the degree of overlap between probability density functions (PDFs), i.e., comparing those PDFs of the mantle for each increment with those of the BSE. Since the errors produced by the Monte Carlo method on the core formation model are lower than those associated to BSE estimates we take the conservative approach of evaluating the increments of late veneer mass using the largest errors. Successful cases were those with an overlap degree higher than one standard deviation and with statistically similar means (overlap $\geq 1\ \sigma$, $p \geq 0.05$). The combination of both statistical analyses on the 5 elements evaluated here (Re, Au, Ir, Os, and Pd) should give strong constraints on the mass of late veneer allowed to match BSE abundances.

Here we find that our model could match Ir and Os abundances in BSE by incorporating 0.2–0.25 wt.% CI-like late veneer, while any addition of late veneer would over-enrich the BSE in Re, Pd and Au (table S7, fig. S7). A simple average for all cases suggests that a maximum 0.09±0.10 wt.% of CI-like late veneer mass is allowed after core formation (table S7), implying that if a late veneer event occurred it was, at most, limited in mass.

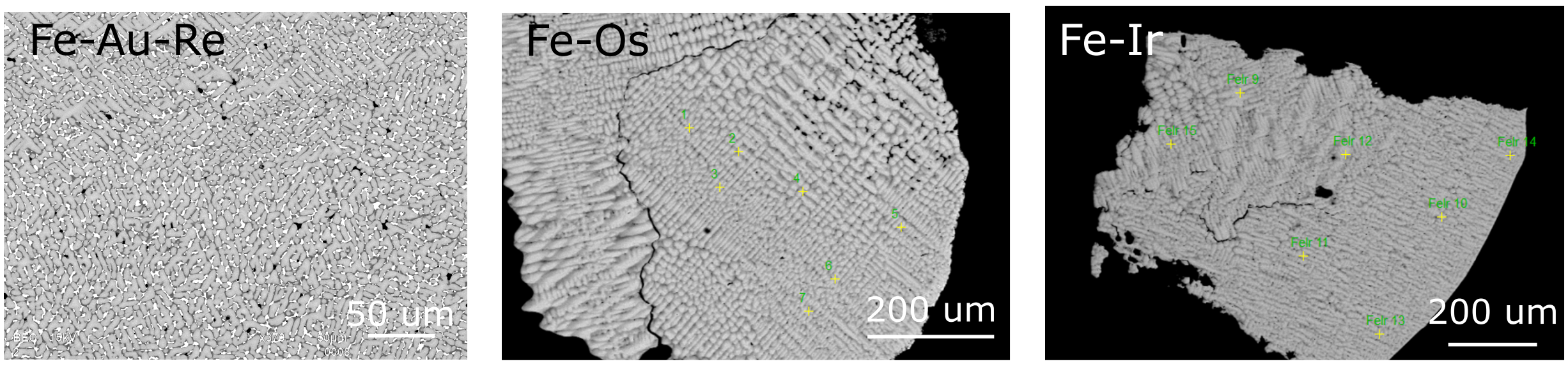

Fig. S1. Backscattered images of three of the starting alloys (Fe-Au-Re, Fe-Os and Fe-Ir) made using piston-cylinder press (2 GPa, 2073 K). The black dots are holes, and yellow crosses are point analysis.

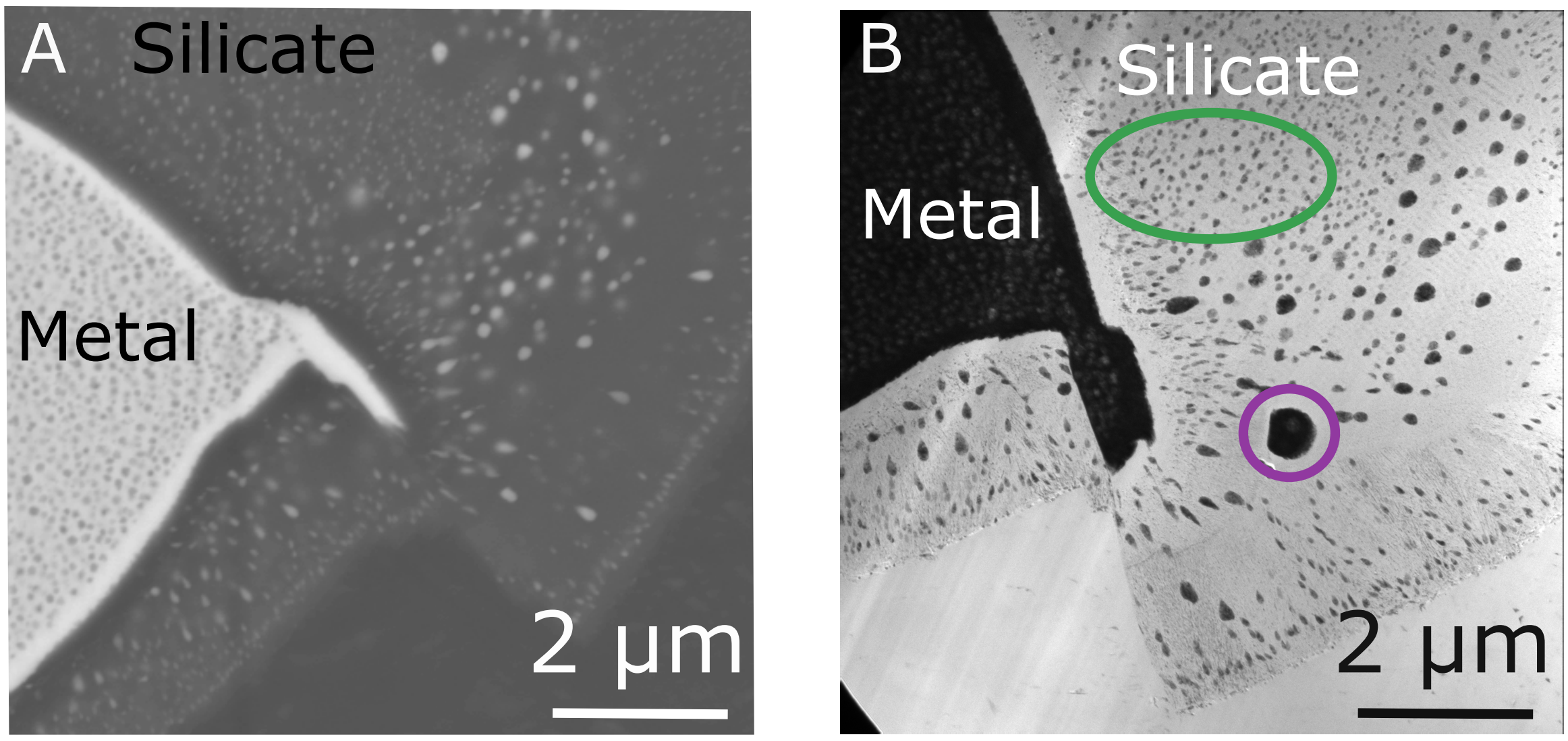

Fig. S2. A) Backscattered electron image of sample FeIr1 synthesized at 70 GPa and 4500 K and B) Transmission electron image of the same sample. These pictures highlight the presence of a diverse population of nuggets in the silicate phase. The metallic inclusion circled with purple in B has a composition similar to the main metallic bleb, whereas the much smaller ones highlighted in green have a different composition, representative of the silicate.

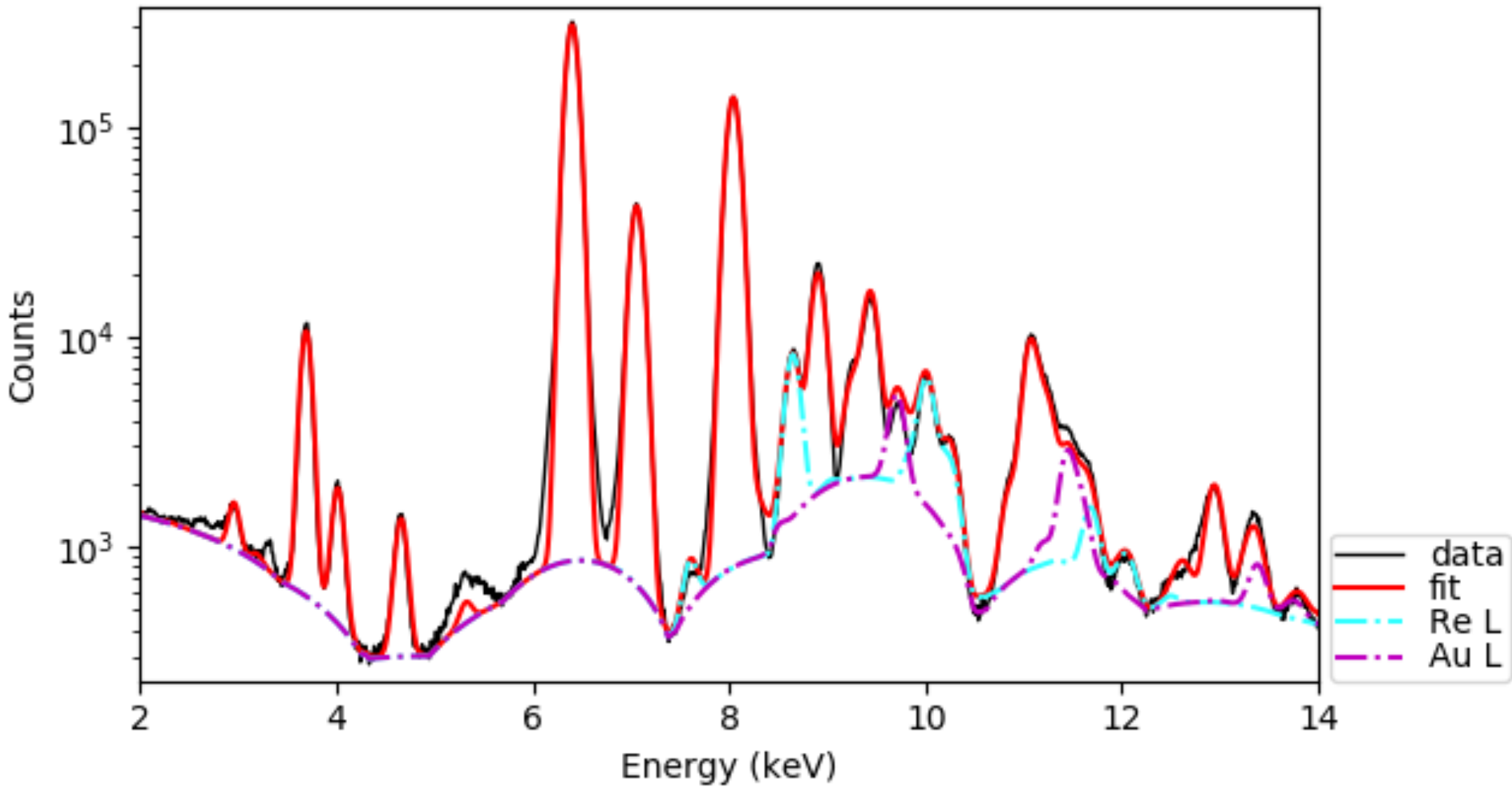

Fig. S3. XRF spectra obtained for sample ESRF. The different lines represent the raw data (black line), the fit obtained with PyMca (red line) and the L lines of Re (cyan line) and Au (purple line).

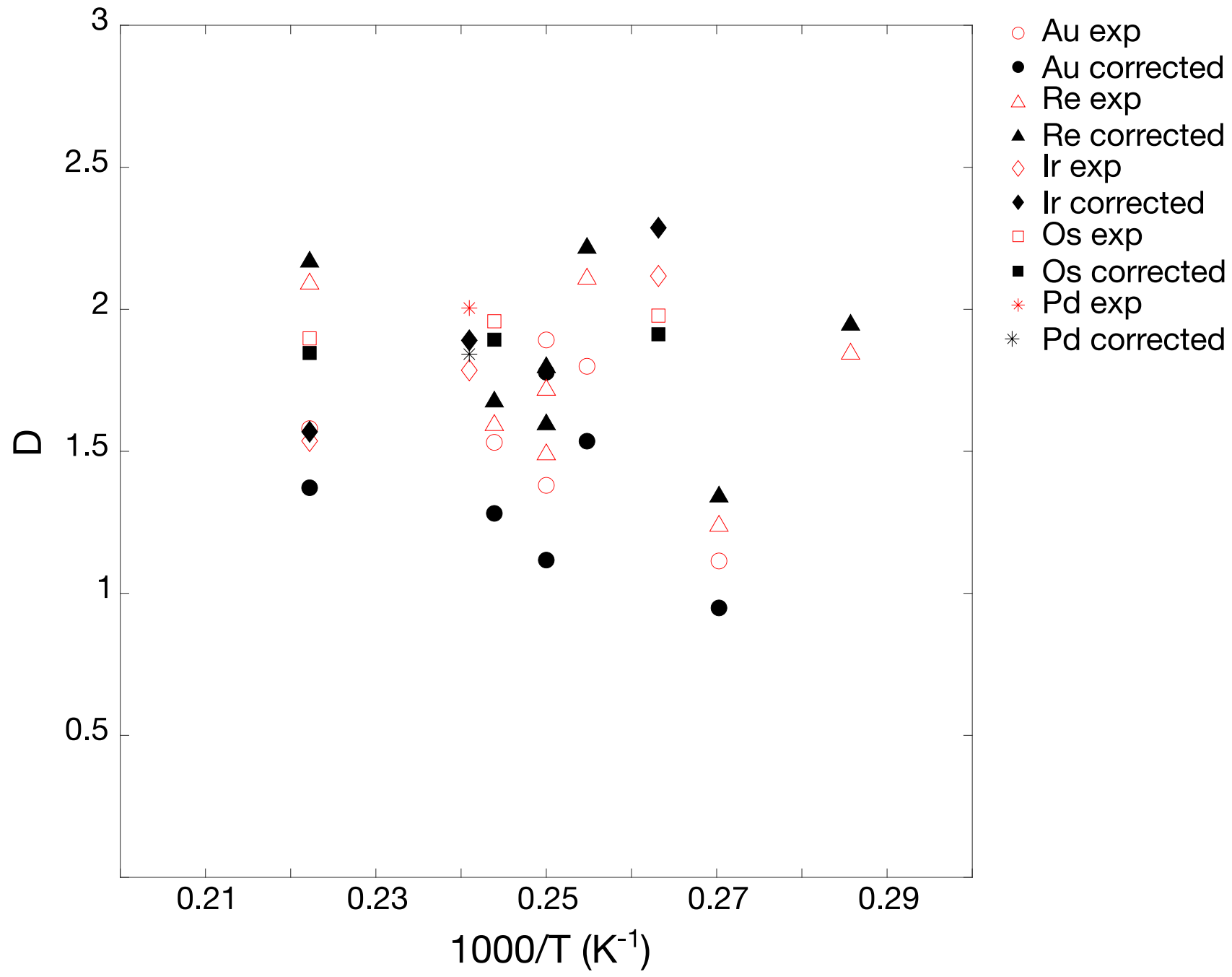

Fig. S4. Comparison between metal-silicate partition coefficients directly measured from experiments (*D*, in moles), and the one recalculated to infinite dilution (D^0) following Eq. S2 and the parameters presented in table S4.

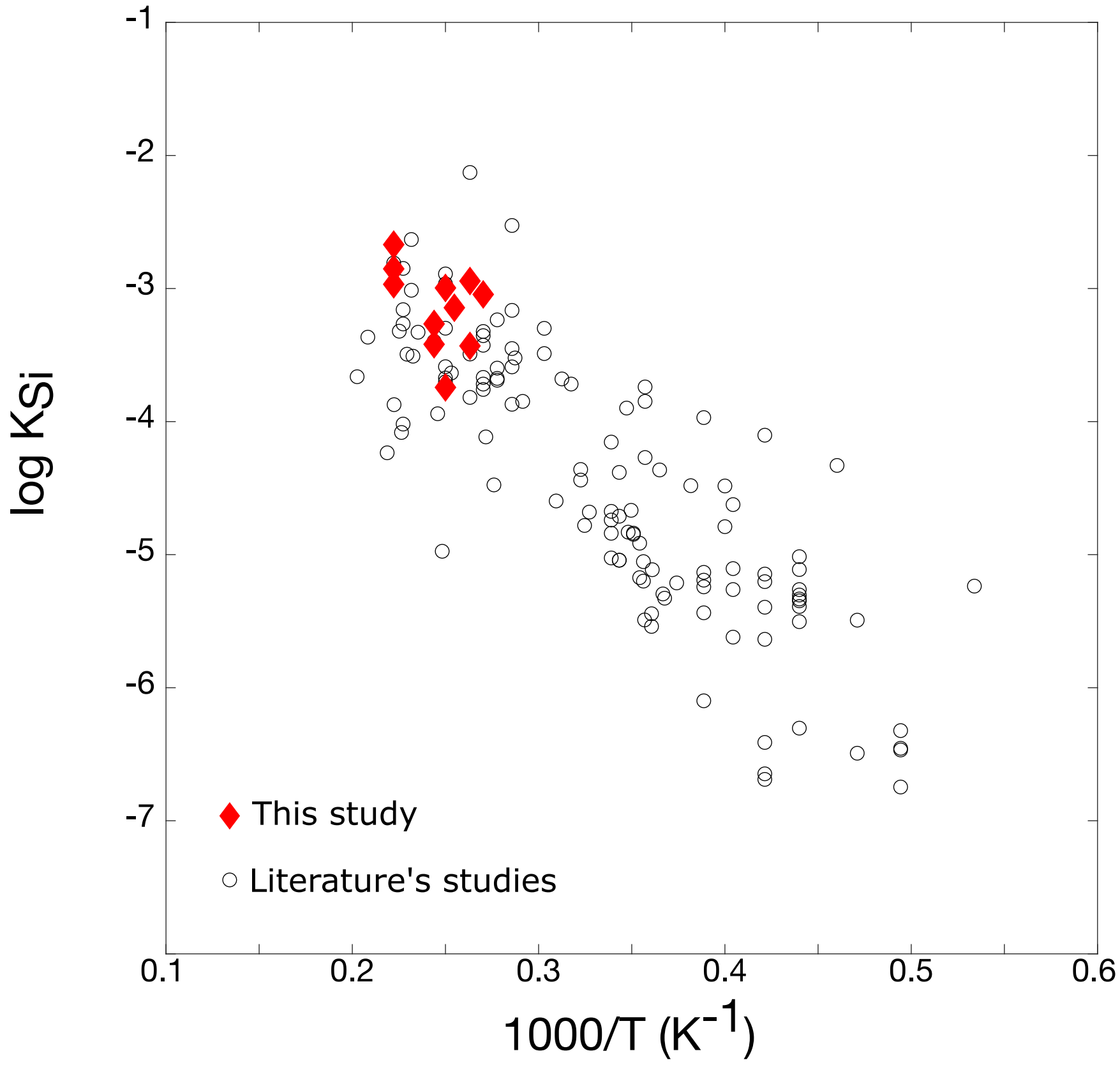

Fig. S5. Equilibrium constant of silicon as a function of inverse temperature. We compile data obtained from large volume presses *(17, 18, 107-111)* and LH-DAC *(67, 78, 112-115)* and this study.

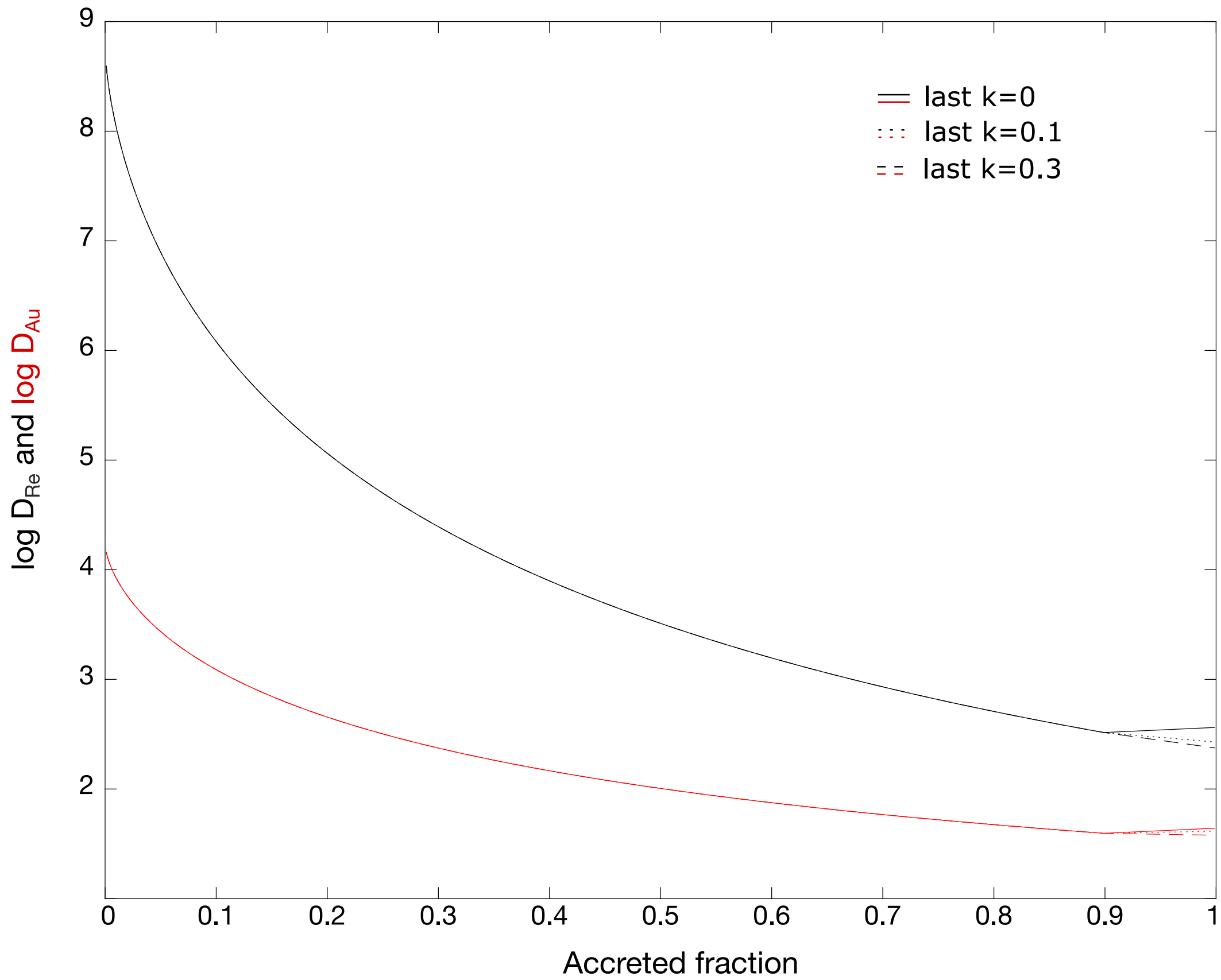

Fig. S6: Evolution of the partition coefficient of Re and Au with the accreted fraction. We present the effect of changing the equilibration of the last giant impact (moon forming impact) on the partitioning of Re (black curve) and Au (red curve).

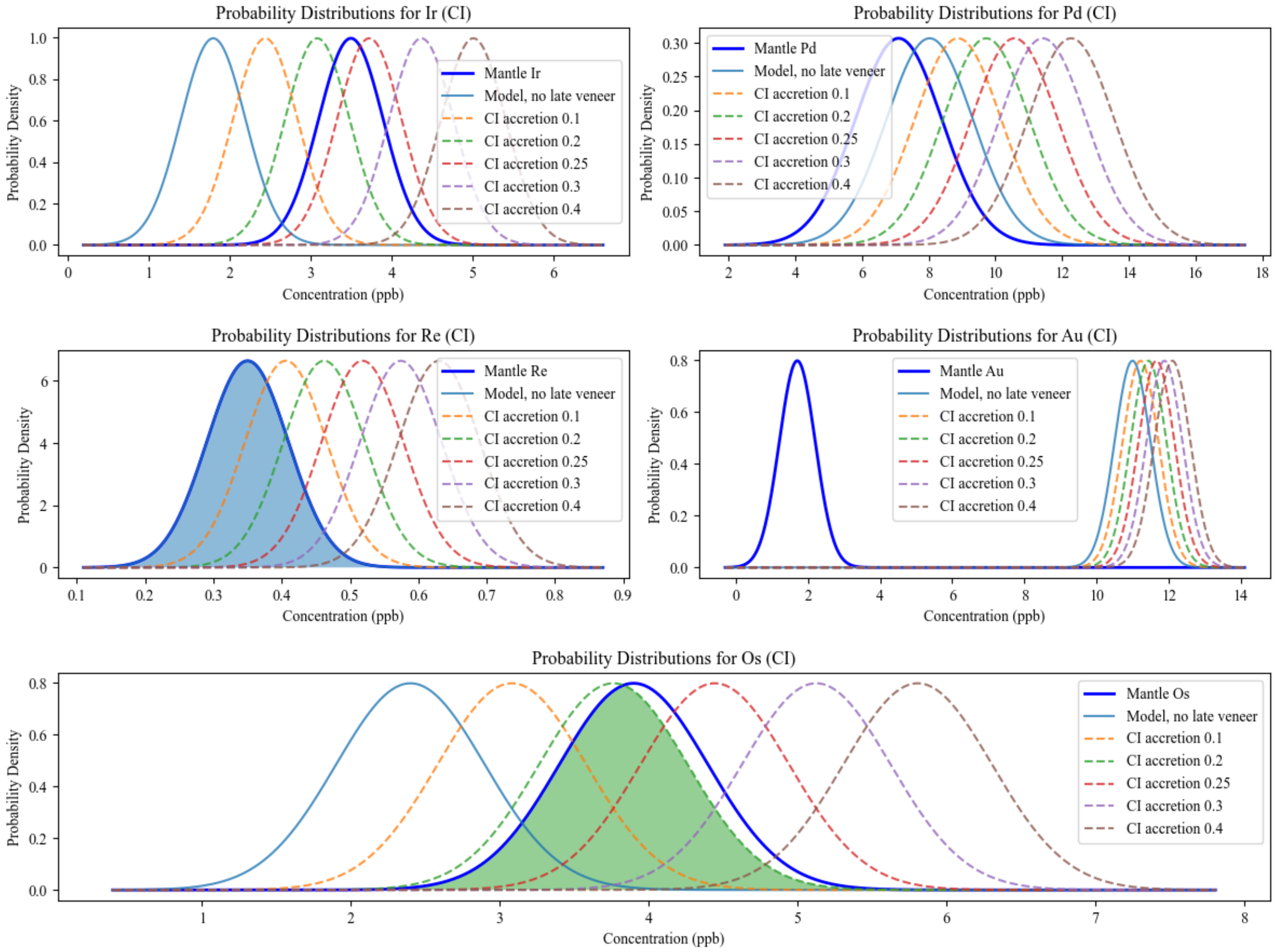

Fig. S7. Probability density functions (PDFs) for HSEs concentrations of each of the 5 elements (Ir, Pd, Re, Au, and Os). Continuous blue curve represents the BSE concentrations, while continuous grey our model results, and dashed colored lines represent 0.1 wt.% increments of CI-like mass to our model up to a maximum of 0.5 wt.%, with an intermediate 0.25 wt.% step. Successful cases for statistical analyses are highlighted as shaded areas (see supplementary text). Numerical results are presented in table S7.

**Samples**	**MORB**	**P1**	**P3**
wt.%	*N=10*	*N=10*	*N=10*
SiO_2	52.07 (0.13)	45.75 (0.12)	46.42 (0.14)
Na_2O	2.19 (0.05)	-	-
CaO	10.85 (0.04)	3.85 (0.04)	3.74 (0.03)
FeO	11.33 (0.09)	7.84 (0.07)	8.23 (0.08)
Al_2O_3	14.57 (0.10)	4.10 (0.07)	4.02 (0.06)
MgO	7.38 (0.05)	38.75 (0.23)	38.03 (0.17)
Total	98.38 (0.17)	100.30 (0.25)	100.44 (0.20)

Table S1. Composition of the starting silicate

MET1		MET2		MET3		MET4	
N=50		*N = 10*		*N=8*		*N=56*	
Fe	60.48 (2.91)	Fe	70.82 (1.55)	Fe	78.29 (0.3)	Fe	56.69 (1.87)
Au	4.82 (2.77)	Os	29.18 (1.55)	Ir	21.71 (0.30)	Ir	30.40 (5.01)
Re	33.71 (5.13)					Pd	11.81 (3.07)
Total	99.05 (0.47)	100		100		98.93 (0.47)	

Table S2. Composition of the starting metal.

Sample	ESRF	BAS_2_60_3	BAS_2_60_2	AuRe2	AuRe3	AuRe4	AuRe6
SMS	MORB	MORB	MORB	MORB	MORB	MORB	MORB
SMM	MET1	MET1	MET1	MET1	MET1	MET1	MET1
P (GPa)	49	72	71	81	55	69	50
T (K)	4000	4500	3925	4100	3700	4000	3500
IW	-1.46	-1.22	-0.67	-1.27	-1.17	-1.06	-0.82
D_{Au}	78	38	63	34	13	24	-
D_{Au}^0	60	23	34	19	9	13	-
D_{Re}	52	123	128	39	17	31	70
D_{Re}^0	62	147	164	47	22	39	88

Sample	FeIr1	FeIr2	FeOs1	FeOs2	FeOs3	PdIr1
SMS	P3	P3	P1	P1	P1	MORB
SMM	MET3	MET3	MET2	MET2	MET2	MET4
P (GPa)	70	42	70	55	40	54
T (K)	4500	3800	4500	4100	3800	4150
IW	-1.3	-1.05	-1.26	-1.61	-1.14	-1.36
D_{Ir}	34	131	-	-	-	61
D_{Ir}^0	37	194	-	-	-	77
D_{Os}	-	-	79	91	95	-
D_{Os}^0	-	-	70	78	82	-
D_{Pd}	-	-	-	-	-	101
D_{Pd}^0	-	-	-	-	-	70

Table S3. Summary of experimental run conditions. Uncertainties on P and T are 5 GPa and 300 K respectively. SMS and SMM refer to the starting material used for the silicate and metal part respectively. D_X is the molar partition coefficient of an element X, and D_X^0 is the molar partition coefficient recalculated at infinite dilution following (*18*, *22*).

Run #	ESRF	BAS_2_60_3	BAS_2_60_2	AuRe2	AuRe3	AuRe4	AuRe6
Silicate phase wt.%	*N=5*	*N=13*	*N=5*	*N=4*	*N=4*	*N=6*	*N=6*
SiO_2	44.53 (1.34)	41.49 (1.82)	34.26 (2.45)	38.11 (1.38)	39.78 (1.50)	38.81 (1.34)	34.71 (0.79)
MgO	11.57 (0.52)	10.13 (0.21)	7.72 (0.52)	8.93 (0.31)	10.40 (0.36)	9.22 (0.45)	7.25 (0.28)
Al_2O_3	15.56 (1.44)	19.19 (0.52)	17.14 (0.99)	26.03 (0.47)	19.36 (0.57)	22.58 (1.09)	14.36 (0.46)
CaO	6.94 (0.94)	6.92 (0.50)	4.34 (1.35)	3.82 (0.16)	5.89 (1.34)	4.48 (0.57)	4.14 (0.26)
Na_2O	3.89 (0.24)	2.45 (0.14)	2.29 (0.16)	2.32 (0.07)	2.92 (0.09)	2.91 (0.16)	2.52 (0.07)
FeO	17.51 (2.09)	19.66 (1.83)	34.24 (5.13)	19.38 (0.73)	20.68 (1.57)	21.24 (2.51)	36.98 (1.63)
Au (ppm)[a]	1300	2800	1000	1500	2000	3800	200
Re (ppm)[a]	3900	1200	2800	5000	10 000	6000	3500
Total	100	100*	100*	100*	100*	100*	100*
metal wt.%	*N=7*	*N=5*	*N=3*	*N=5*	*N=4*	*N=6*	*N=5*
Fe	66.75 (0.71)	59.57 (0.28)	55.57 (0.21)	64.78 (0.54)	65.44 (0.61)	56.29 (1.27)	69.53 (0.75)
Si	2.10 (0.08)	4.96 (0.69)	2.02 (0.15)	3.11 (0.12)	6.88 (0.28)	6.01 (0.44)	0.00 (0.00)
O	4.30 (0.16)	4.93 (0.56)	6.45 (0.25)	5.93 (0.26)	5.93 (0.64)	6.63 (1.22)	3.53 (0.41)
Au	8.63 (0.09)	11.61 (0.22)	6.25 (0.22)	5.75 (0.09)	3.77 (0.28)	11.33 (0.27)	1.69 (0.09)
Re	17.98 (0.25)	17.87 (0.48)	29.60 (0.52)	20.23 (0.62)	17.88 (0.54)	19.14 (0.19)	25.25 (1.09)
Total	100	100*	100*	100*	100*	100*	100

Run #	FeIr1	FeIr2	FeOs1	FeOs2	FeOs3	PdIr1
Silicate phase wt.%	*N=9*	*N=18*	*N=9*	*N=8*	*N=11*	*N=3*
SiO_2	36.80 (0.89)	36.91 (1.66)	35.27 (1.02)	39.10 (1.05)	37.36 (0.68)	42.65 (0.62)
MgO	34.52 (0.97)	29.24 (0.66)	34.27 (0.88)	35.89 (0.67)	30.35 (0.79)	9.48 (0.92)
Al_2O_3	6.44 (0.33)	5.32 (0.13)	6.82 (0.23)	6.47 (0.25)	4.70 (0.18)	17.06 (2.17)
CaO	3.90 (0.14)	3.62 (0.13)	3.75 (0.19)	4.07 (0.25)	4.20 (0.18)	9.35 (3.06)
Na_2O	-	-	-	-	-	2.39 (0.73)
FeO	18.24 (1.71)	24.76 (2.20)	19.72 (1.06)	14.46 (1.28)	23.32 (1.48)	16.32 (1.50)
Ir (ppm)[a]	2500	1557	-	-	-	
Os (ppm)[a]	-	-	2300	2700	6000	3500
Pd (ppm)[a]	-	-	-	-	-	1100
Total	100*	100*	100*	100*	100*	100*
metal wt.%	*N=5*	*N=5*	*N=6*	*N=6*	*N=6*	*N=6*
Fe	73.17 (1.29)	66.83 (1.12)	68.37 (1.45)	67.59 (1.82)	51.82 (1.87)	58.71 (0.27)
Si	10.30 (0.49)	5.52 (0.73)	6.44 (0.24)	5.40 (0.26)	1.64 (0.19)	6.72 (0.11)
O	6.21 (1.12)	5.25 (1.10)	5.59 (0.42)	3.74 (0.35)	2.90 (0.32)	3.29 (0.2)
Ir	9.72 (0.32)	21.25 (0.71)	-	-	-	20.68 (0.11)
Os	-	-	19.21 (1.18)	22.95 (1.85)	43.54 (1.98)	-
Pd	-	-	-	-	-	10.59 (0.13)
Al	0.24 (0.10)	0.63 (0.05)	0.16 (0.10)	b.d.	b.d.	b.d.
Mg	0.35 (0.15)	0.47 (0.03)	0.23 (0.13)	b.d.	b.d.	b.d.
Total	100*	100*	100*	100*	100*	100*

Table S4. Composition of the experimental runs. Major elements were acquired using FEG-EDS, whereas trace elements in the silicate phase ([a]) were acquired using nano-XRF at the ESRF synchrotron. * indicates the presence of Cu measured because of the fluorescence of the TEM grid, but not reported for clarity. B.d. stands for below detection limit.

Standard IZ	Expected values (ppm)	Measured values (ppm)	Measured/Expected
Ru	994	909	0.91
Rh*	65	57	0.88
Pd*	45	25	0.56
Ag	1231	1094	0.89
Re	1198	1035	0.86
Os*	6	25	4.17
Ir	423	161	0.38

Table S5 Comparison between composition of standard IZ (*85*) and measurements by nano-XRF (this study). *Elements for which abundances are expected to be below 100 ppm are not well resolved.

Run #	$W^{H, 1bar}$	W^{S}	W^{V}	T^{0}(K)
Fe-Re	-19949	0	0	2700
Re-Fe	-19949	0	0	2700
Fe-Au	5498	63.9	0.15	1873
Au-Fe	5498	63.9	0.15	1873
Re-Au	23800	0	0	2700
Au-Re	23800	0	0	2700
Fe-Pd	5498	63.9	0.15	1873
Pd-Fe	5498	63.9	0.15	1873
Pd-Ir	30751	3	0	2800
Ir-Pd	30751	3	0	2800
Fe-Ir	-59360	0	0.08	2720
Ir-Fe	-63360	0	0.08	2720

Table S6. Margules parameters W used in this study for Fe-HSE and HSE-HSE binaries, from (*18*, *27*). For Os, we used the values proposed by (*90*) such as WOsFe=WFeOs=-10883.

Element	Mantle (ppb)(*25*, *26*)	Model (ppb)	Element added per increment (ppb)	Mass Increment (wt.%)	Overlap	t-stat	p-value
Ir	3.5±0.4	1.9±0.4	0.642	0	0.03	45.65	2.58E-68
Ir	3.5±0.4	1.9±0.4	0.642	0.1	0.19	27.85	4.96E-49
Ir	3.5±0.4	1.9±0.4	0.642	0.2	0.60	10.1	3.73E-17
Ir	3.5±0.4	1.9±0.4	0.642	0.25	0.78	-7.23	6.38E-11
Ir	3.5±0.4	1.9±0.4	0.642	0.3	0.28	-23.84	1.35E-47
Ir	3.5±0.4	1.9±0.4	0.642	0.4	0.06	-39.5	2.19E-75
Pd	7.1±1.3	8.03±1.30	0.848	0	0.72	-9.02	1.49E-14
Pd	7.1±1.3	8.03±1.30	0.848	0.1	0.49	-16.21	1.25E-29
Pd	7.1±1.3	8.03±1.30	0.848	0.2	0.31	-23.37	3.21E-42
Pd	7.1±1.3	8.03±1.30	0.848	0.25	0.18	-30.5	1.49E-52
Pd	7.1±1.3	8.03±1.30	0.848	0.3	0.10	-37.57	2.58E-61
Pd	7.1±1.3	8.03±1.30	0.848	0.4	0.05	-44.57	5.14E-69
Re	0.35±0.06	0.35±0.06	0.056	0	1.00	-1.14	0.26
Re	0.35±0.06	0.35±0.06	0.056	0.1	0.64	-11.43	8.06E-20
Re	0.35±0.06	0.35±0.06	0.056	0.2	0.35	-21.64	1.08E-39
Re	0.35±0.06	0.35±0.06	0.056	0.25	0.16	-31.7	4.92E-55
Re	0.35±0.06	0.35±0.06	0.056	0.3	0.06	-41.55	1.12E-67
Re	0.35±0.06	0.35±0.06	0.056	0.4	0.02	-51.13	6.37E-79
Au	1.7±0.5	11.0±0.5	0.221	0	<0.01	-205.96	3.76E-132
Au	1.7±0.5	11.0±0.5	0.221	0.1	<0.01	-210.51	8.96E-134
Au	1.7±0.5	11.0±0.5	0.221	0.2	<0.01	-214.39	1.27E-136
Au	1.7±0.5	11.0±0.5	0.221	0.25	<0.01	-217.58	1.05E-140
Au	1.7±0.5	11.0±0.5	0.221	0.3	<0.01	-220.08	5.30E-146
Au	1.7±0.5	11.0±0.5	0.221	0.4	<0.01	-221.91	1.87E-152
Os	3.9±0.5	2.4±0.5	0.682	0	0.13	31.89	7.04E-54
Os	3.9±0.5	2.4±0.5	0.682	0.1	0.41	16.82	6.57E-31
Os	3.9±0.5	2.4±0.5	0.682	0.2	0.89	1.81	0.07
Os	3.9±0.5	2.4±0.5	0.682	0.25	0.59	-12.91	1.22E-23
Os	3.9±0.5	2.4±0.5	0.682	0.3	0.22	-27.15	7.26E-52
Os	3.9±0.5	2.4±0.5	0.682	0.4	0.06	-40.74	1.24E-73
	Average late veneer mass allowed for all elements			0.09±0.11			

Table S7. Results for statistical evaluation of late veneer mass addition after core formation on Earth. Average late veneer mass rises from a simple average from statistically best scenarios for each element.

REFERENCES

1. A. Borisov, H. Palme, B. Spettel, Solubility of palladium in silicate melts: Implications for core formation in the Earth. *Geochim. Cosmochim. Acta* **58**, 705–716 (1994).

2. A. Borisov, H. Palme, Experimental determination of the solubility of Au in silicate melts. *Mineral. Petrol.* **56**, 297–312 (1996).

3. A. Borisov, H. Palme, The solubility of iridium in silicate melts: New data from experiments with $lr_{10}Pt_{90}$ alloys. *Geochim. Cosmochim. Acta* **59**, 481–485 (1995).

4. H. S. C. O'Neill, D. B. Dingwell, A. Borisov, B. Spettel, H. Palme, Experimental petrochemistry of some highly siderophile elements at high temperatures, and some implications for core formation and the mantle's early history. *Chem. Geol.* **120**, 255–273 (1995).

5. C.-L. Chou, D. M. Shaw, J. H. Crocker, Siderophile trace elements in the Earth' oceanic crust and upper mantle. *J. Geophys. Res.* **88**, A507–A518 (1983).

6. K. Kimura, R. S. Lewis, E. Anders, Distribution of gold and rhenium between nickel-iron and silicate melts: Implications for the abundance of siderophile elements on the Earth and Moon. *Geochim. Cosmochim. Acta* **38**, 683–701 (1974).

7. K. K. Turekian, S. P. Clark Jr., Inhomogeneous accumulation of the earth from the primitive solar nebula. *Earth Planet. Sci. Lett.* **6**, 346–348 (1969).

8. H. Wänke, Constitution of terrestrial planets. *Philos. Trans. R. Soc.* **303**, 287–302 (1981).

9. F. Albarède, Volatile accretion history of the terrestrial planets and dynamic implications. *Nature* **461**, 1227–1233 (2009).

10. Z. Wang, H. Becker, Ratios of S, Se and Te in the silicate Earth require a volatile-rich late veneer. *Nature* **499**, 328–331 (2013).

11. R. J. Walker, *Siderophile Elements in Tracing Planetary Formation and Evolution* (Geochemica., 2016), vol. 5.

12. J. Siebert, J. Badro, D. Antonangeli, F. J. Ryerson, Metal–silicate partitioning of Ni and Co in a deep magma ocean. *Earth Planet. Sci. Lett.* **321**, 189–197 (2012).

13. J. Wade, B. J. Wood, Core formation and the oxidation state of the Earth. *Earth Planet. Sci. Lett.* **236**, 78–95 (2005).

14. W. B. Tonks, H. J. Melosh, Magma ocean formation due to giant impacts. *J. Geophys. Res.* **98**, 5319–5333 (1993).

15. V. R. Murthy, Early differentiation of the earth and the problem of mantle siderophile elements: A new approach. *Science* **253**, 303–306 (1991).

16. T.-A. Suer, J. Siebert, L. Remusat, J. M. D. Day, S. Borensztajn, B. Doisneau, G. Fiquet, Reconciling metal–silicate partitioning and late accretion in the Earth. *Nat. Commun.* **12**, 2913 (2021).

17. R. A. Fischer, Y. Nakajima, A. J. Campbell, D. J. Frost, D. Harries, F. Langenhorst, N. Miyajima, K. Pollok, D. C. Rubie, High pressure metal–silicate partitioning of Ni, Co, V, Cr, Si, and O. *Geochim. Cosmochim. Acta* **167**, 177–194 (2015).

18. U. Mann, D. J. Frost, D. C. Rubie, H. Becker, A. Audétat, Partitioning of Ru, Rh, Pd, Re, Ir and Pt between liquid metal and silicate at high pressures and high temperatures - Implications for the origin of highly siderophile element concentrations in the Earth's mantle. *Geochim. Cosmochim. Acta* **84**, 593–613 (2012).

19. J. M. Brenan, W. F. McDonough, Core formation and metal–silicate fractionation of osmium and iridium from gold. *Nat. Geosci.* **2**, 798–801 (2009).

20. N. R. Bennett, J. M. Brenan, Controls on the solubility of rhenium in silicate melt: Implications for the osmium isotopic composition of Earth's mantle. *Earth Planet. Sci. Lett.* **361**, 320–332 (2013).

21. I. Blanchard, D. C. Rubie, E. S. Jennings, I. A. Franchi, X. Zhao, S. Petitgirard, N. Miyajima, S. A. Jacobson, A. Morbidelli, The metal–silicate partitioning of carbon during Earth's

accretion and its distribution in the early solar system. *Earth Planet. Sci. Lett.* **580**, 117374 (2022a).

22. B. Mukhopadhyay, S. Basu, M. J. Holdaway, A discussion of Margules-type formulations for multicomponent solutions with a generalized approach. *Geochim. Cosmochim. Acta* **57**, 277–283 (1993).

23. S. N. Raymond, H. E. Schlichting, F. Hersant, F. Selsis, Dynamical and collisional constraints on a stochastic late veneer on the terrestrial planets. *Icarus* **226**, 671–681 (2013).

24. H. Becker, M. F. Horan, R. J. Walker, S. Gao, J.-P. Lorand, R. L. Rudnick, Highly siderophile element composition of the Earth's primitive upper mantle: Constraints from new data on peridotite massifs and xenoliths. *Geochim. Cosmochim. Acta* **70**, 4528–4550 (2006).

25. J. M. D. Day, A. D. Brandon, R. J. Walker, "Highly siderophile elements in Earth, Mars, the Moon, and Asteroids" in *Highly Siderophile and Strongly Chalcophile Elements in High-Temperature Geochemistry and Cosmochemistry* (De Gruyter, 2016), pp. 161–238.

26. W. Ertel, H. S. C. O'Neill, P. J. Ylvester, D. B. Ingwell, B. Pettel, The solubility of rhenium in silicate melts: Implications for the geochemical properties of rhenium at high temperatures. *Geochim. Cosmochim. Acta* **65**, 2161–2170 (2001).

27. A. Borisov, H. Palme, Solubilities of noble metals in Fe-containing silicate melts as derived from experiments in Fe-free systems. *Am. Mineral.* **85**, 1665–1673 (2000).

28. W. Ertel, H. S. C. O'Neill, P. J. Sylvester, D. B. Dingwell, Solubilities of Pt and Rh in a haplobasaltic silicate melt at 1300°C. *Geochim. Cosmochim. Acta* **63**, 2439–2449 (1999).

29. I. S. Puchtel, J. Blichert-Toft, M. F. Horan, M. Touboul, R. J. Walker, The komatiite testimony to ancient mantle heterogeneity. *Chem. Geol.* **594**, 120776 (2022).

30. I. S. Puchtel, M. Touboul, J. Blichert-Toft, R. J. Walker, A. D. Brandon, R. W. Nicklas, V. S. Kulikov, A. V. Samsonov, Lithophile and siderophile element systematics of Earth's mantle at the Archean-Proterozoic boundary: Evidence from 2.4 Ga komatiites. *Geochim. Cosmochim. Acta* **180**, 227–255 (2016).

31. I. S. Puchtel, J. Blichert-Toft, M. Touboul, R. J. Walker, ^{182}W and HSE constraints from 2.7 Ga komatiites on the heterogeneous nature of the Archean mantle. *Geochim. Cosmochim. Acta* **228**, 1–26 (2018).

32. I. S. Puchtel, A. Mundl-Petermeier, M. Horan, E. J. Hanski, J. Blichert-Toft, R. J. Walker, Ultra-depleted 2.05 Ga komatiites of Finnish Lapland: Products of grainy late accretion or core-mantle interaction? *Chem. Geol.* **554**, 119801 (2020).

33. M. Touboul, I. S. Puchtel, R. J. Walker, ^{182}W evidence for long-term preservation of early mantle differentiation products. *Science* **335**, 1065–1069 (2012).

34. P. Waterton, J. Mungall, D. G. Pearson, The komatiite-mantle platinum-group element paradox. *Geochim. Cosmochim. Acta* **313**, 214–242 (2021).

35. N. Messling, M. Willbold, L. Kallas, T. Elliott, J. G. Fitton, T. Müller, D. Geist, Ru and W isotope systematics in ocean island basalts reveals core leakage. *Nature* **642**, 376–380 (2025).

36. M. Fischer-Gödde, B. M. Elfers, C. Münker, K. Szilas, W. D. Maier, N. Messling, T. Morishita, M. Van Kranendonk, H. Smithies, Ruthenium isotope vestige of Earth's pre-late-veneer mantle preserved in Archaean rocks. *Nature* **579**, 240–244 (2020).

37. R. J. Anslow, M. Landeau, A. Bonsor, J. Itcovitz, O. Shorttle, The efficient delivery of highly siderophile elements to the core creates a mass accretion catastrophe for the Earth. *J. Geophys. Res. Planets* **131**, e2025JE009328 (2026).

38. K. T. Wheeler, D. Walker, W. F. McDonough, Pd and Ag metal-silicate partitioning applied to Earth differentiation and core-mantle exchange. *Meteorit. Planet. Sci.* **46**, 199–217 (2011).

39. K. Righter, K. Pando, M. Humayun, N. Waeselmann, S. Yang, A. Boujibar, L. R. Danielson, Effect of silicon on activity coefficients of siderophile elements (Au, Pd, Pt, P, Ga, Cu, Zn, and Pb) in liquid Fe: Roles of core formation, late sulfide matte, and late veneer in shaping terrestrial mantle geochemistry. *Geochim. Cosmochim. Acta* **232**, 101–123 (2018).

40. N. R. Bennett, J. M. Brenan, Y. Fei, Thermometry of the magma ocean: Controls on the metal-silicate partitioning of gold. *Geochim. Cosmochim. Acta* **184**, 173–192 (2016).

41. D. C. Rubie, V. Laurenz, S. A. Jacobson, A. Morbidelli, H. Palme, A. K. Vogel, D. J. Frost, Highly siderophile elements were stripped from Earth's mantle by iron sulfide segregation. *Science* **353**, 1141–1144 (2016).

42. H. S. C. O'Neill, The origin of the Moon and the early history of the Earth—A chemical model. Part 2: The Earth. 55, 1135–1157 (1991).

43. V. Laurenz, D. C. Rubie, D. J. Frost, A. K. Vogel, The importance of sulfur for the behavior of highly-siderophile elements during Earth's differentiation. *Geochim. Cosmochim. Acta* **194**, 123–138 (2016).

44. E. S. Steenstra, O. T. Lord, S. Vitale, E. S. Bullock, S. Klemme, M. Walter, Sulfur solubility in a deep magma ocean and implications for the deep sulfur cycle. *Geochem. Perspect. Lett.* **22**, 5–9 (2022).

45. I. Blanchard, J. Siebert, E. Kubik, A. Minchenkova, L. Calvo, N. Wehr, Earth's deep magma ocean never reached sulfide saturation. *Geochem. Perspect. Lett.* **34**, 6–10 (2025).

46. D. J. Frost, C. Liebske, F. Langenhorst, C. A. McCammon, R. G. Trønnes, D. C. Rubie, Experimental evidence for the existence of iron-rich metal in the Earth's lower mantle. *Nature* **428**, 409–412 (2004).

47. H. L. Zhang, M. M. Hirschmann, O. T. Lord, A. Rosenthal, S. Yaroslavtsev, E. Cottrell, A. I. Chumakov, M. J. Walter, Ferric iron stabilization at deep magma ocean conditions. *Sci. Adv.* **10**, eadp1752 (2024).

48. R. J. Walker, Highly siderophile elements in the Earth, Moon and Mars: Update and implications for planetary accretion and differentiation. *Geochemistry* **69**, 101–125 (2009).

49. R. J. Walker, M. F. Horan, C. K. Shearer, J. J. Papike, Low abundances of highly siderophile elements in the lunar mantle: Evidence for prolonged late accretion. *Earth Planet. Sci. Lett.* **224**, 399–413 (2004).

50. J. M. D. Day, D. G. Pearson, L. A. Taylor, Highly siderophile element constraints on accretion and differentiation of the Earth-Moon system. *Science* **315**, 217–219 (2007).

51. W. F. Bottke, R. J. Walker, J. M. D. Day, D. Nesvorny, L. Elkins-Tanton, Stochastic late accretion to Earth, the Moon, and Mars. *Science* **330**, 1527–1530 (2010).

52. R. Brasser, S. J. Mojzsis, S. C. Werner, S. Matsumura, S. Ida, Late veneer and late accretion to the terrestrial planets. *Earth Planet. Sci. Lett.* **455**, 85–93 (2016).

53. J. W. Valley, A. J. Cavosie, T. Ushikubo, D. A. Reinhard, D. F. Lawrence, D. J. Larson, P. H. Clifton, T. F. Kelly, S. A. Wilde, D. E. Moser, M. J. Spicuzza, Hadean age for a post-magma-ocean zircon confirmed by atom-probe tomography. *Nat. Geosci.* **7**, 219–223 (2014).

54. J. Tusch, P. Sprung, J. van de Löcht, J. E. Hoffmann, A. J. Boyd, M. T. Rosing, C. Münker, Uniform 182W isotope compositions in Eoarchean rocks from the Isua region, SW Greenland: The role of early silicate differentiation and missing late veneer. *Geochim. Cosmochim. Acta* **257**, 284–310 (2019).

55. R. M. Canup, E. Asphaug, Origin of the Moon in a giant impact near the end of the Earth's formation. *Nature* **412**, 708–712 (2001).

56. M. Landeau, P. Olson, R. Deguen, B. H. Hirsh, Core merging and stratification following giant impact. *Nat. Geosci.* **9**, 786–789 (2016).

57. A. D. Brandon, I. S. Puchtel, R. J. Walker, J. M. D. Day, A. J. Irving, L. A. Taylor, Evolution of the martian mantle inferred from the 187Re-187Os isotope and highly siderophile element abundance systematics of shergottite meteorites. *Geochim. Cosmochim. Acta* **76**, 206–235 (2012).

58. K. Righter, L. R. Danielson, K. M. Pando, J. Williams, M. Humayun, R. L. Hervig, T. G. Sharp, Highly siderophile element (HSE) abundances in the mantle of Mars are due to core formation at high pressure and temperature. *Meteorit. Planet. Sci.* **50**, 604–631 (2015).

59. H. Samuel, M. Drilleau, A. Rivoldini, Z. Xu, Q. Huang, R. F. Garcia, V. Lekić, J. C. E. Irving, J. Badro, P. H. Lognonné, J. A. D. Connolly, T. Kawamura, T. Gudkova, W. B. Banerdt, Geophysical evidence for an enriched molten silicate layer above Mars's core. *Nature* **622**, 712–717 (2023).

60. C. W. Dale, K. W. Burton, R. C. Greenwood, A. Gannoun, J. Wade, B. J. Wood, D. G. Pearson, Late accretion on the earliest planetesimals revealed by the highly siderophile elements. *Science* **336**, 72–75 (2012).

61. J. M. D. Day, R. J. Walker, L. Qin, D. R. Iii, Late accretion as a natural consequence of planetary growth. *Nat. Geosci.* **5**, 614–617 (2012).

62. A. N. Halliday, The origins of volatiles in the terrestrial planets. *Geochim. Cosmochim. Acta* **105**, 146–171 (2013).

63. B. J. Wood, A. N. Halliday, M. Rehkämper, Volatile accretion history of the Earth. *Nature* **467**, 2009–2010 (2010).

64. J. Labidi, P. Cartigny, M. Moreira, Non-chondritic sulphur isotope composition of the terrestrial mantle. *Nature* **501**, 208–211 (2013).

65. W. Wang, M. J. Walter, J. P. Brodholt, S. Huang, M. I. Petaev, Chalcogen isotopes reveal limited volatile contribution from late veneer to Earth. *Sci. Adv.* **9**, eadh0670 (2023).

66. A.-L. Auzende, J. Gillot, A. Coquet, L. Hennet, G. Ona-Nguema, D. Bonnin, I. Esteve, M. Roskosz, G. Fiquet, Synthesis of amorphous MgO-rich peridotitic starting material for laser-heated diamond anvil cell experiments–application to iron partitioning in the mantle. *High Press. Res.* **31**, 199–213 (2011).

67. I. Blanchard, J. Siebert, S. Borensztajn, J. Badro, The solubility of heat-producing elements in Earth's core. *Geochem. Persp. Lett.* **5**, 1–5 (2017).

68. S. S. Lobanov, L. Schifferle, R. Schulz, Gated detection of supercontinuum pulses enables optical probing of solid and molten silicates at extreme pressure-temperature conditions. *Rev. Sci. Instrum.* **91**, 053103 (2020).

69. J. A. Montoya, A. F. Goncharov, Finite element calculations of the time dependent thermal fluxes in the laser-heated diamond anvil cell. *J. Appl. Phys.* **111**, 112617 (2012).

70. R. Fréville, A. Dewaele, N. Guignot, P. Faure, L. Henry, G. Garbarino, M. Mezouar, High-pressure-high-temperature phase diagram of tin. *Phys. Rev. B* **109**, 104116 (2024).

71. L. R. Benedetti, P. Loubeyre, Temperature gradients, wavelength-dependent emissivity, and accuracy of high and very-high temperatures measured in the laser-heated diamond cell. *High Press. Res.* **24**, 423–445 (2004).

72. Y. Akahama, H. Kawamura, High-pressure Raman spectroscopy of diamond anvils to 250 GPa: Method for pressure determination in the multimegabar pressure range. *J. Appl. Phys.* **96**, 3748–3751 (2004).

73. D. Andrault, N. Bolfan-Casanova, G. L. Nigro, M. A. Bouhifd, G. Garbarino, M. Mezouar, Solidus and liquidus profiles of chondritic mantle: Implication for melting of the Earth across its history. *Earth Planet. Sci. Lett.* **174**, 181–191 (2011).

74. G. Fiquet, A. L. Auzende, J. Siebert, A. Corgne, H. Bureau, H. Ozawa, G. Garbarino, Melting of peridotite to 140 gigapascals. *Science* **329**, 1516–1518 (2010).

75. R. Boehler, High-pressure experiments and the phase diagram of lower mantle and core materials. *Rev. Geophys.* **38**, 221–245 (2000).

76. I. Blanchard, S. Petitgirard, V. Laurenz, N. Miyajima, M. Wilke, D. C. Rubie, S. S. Lobanov, L. Hennet, W. Morgenroth, R. Tucoulou, V. Bonino, X. Zhao, I. A. Franchi, Chemical analysis of trace elements at the nanoscale in samples recovered from laser-heated diamond anvil cell experiments. *Phys. Chem. Miner.* **49**, 18 (2022b).

77. E. S. Jennings, J. Wade, V. Laurenz, S. Petitgirard, Diamond anvil cell partitioning experiments for accretion and core formation: Testing the limitations of electron microprobe analysis. *Microsc. Microanal.* **25**, 1–10 (2019).

78. B. A. Chidester, Z. Rahman, K. Righter, A. J. Campbell, Metal – silicate partitioning of U: Implications for the heat budget of the core and evidence for reduced U in the mantle. *Geochim. Cosmochim. Acta* **199**, 1–12 (2017).

79. V. Malavergne, E. Charon, J. Jones, P. Cordier, K. Righter, D. Deldicque, L. Hennet, The formation of nuggets of highly siderophile elements in quenched silicate melts at high temperatures: Before or during the silicate quench? *Earth Planet. Sci. Lett.* **434**, 197–207 (2016).

80. W. Ertel, D. B. Dingwell, P. J. Sylvester, Siderophile elements in silicate melts – A review of the mechanically assisted equilibration technique and the nanonugget issue. *Chem. Geol.* **248**, 119–139 (2008).

81. E. Cottrell, D. Walker, Constraints on core formation from Pt partitioning in mafic silicate liquids at high temperatures. *Geochim. Cosmochim. Acta* **70**, 1565–1580 (2006).

82. E. Médard, M. W. Schmidt, M. Wälle, N. S. Keller, D. Günther, Platinum partitioning between metal and silicate melts: Core formation, late veneer and the nanonuggets issue. *Geochim. Cosmochim. Acta* **162**, 183–201 (2015).

83. D. Huang, J. Siebert, P. Sossi, E. Kubik, G. Avice, M. Murakami, Nitrogen sequestration in the core at megabar pressure and implications for terrestrial accretion. *Geochim. Cosmochim. Acta* **376**, 100–112 (2024).

84. V. A. Solé, E. Papillon, M. Cotte, P. Walter, J. Susini, A multiplatform code for the analysis of energy-dispersive X-ray fluorescence spectra. *Spectrochim. Acta Part B At. Spectrosc.* **62**, 63–68 (2007).

85. J. Chen, G. Mallmann, I. Zhukova, H. O'Neill, Development of PGE-bearing silicate glass standards for quantitative trace element analysis in silicate-based metallurgical slags. *J. Sustainable Metall.* **6**, 691–699 (2020).

86. K. Righter, M. Humayun, L. Danielson, Partitioning of palladium at high pressures and temperatures during core formation. *Nat. Geosci.* **1**, 321–323 (2008).

87. T. Yokoyama, D. Walker, R. J. Walker, Low osmium solubility in silicate at high pressures and temperatures. *Earth Planet. Sci. Lett.* **279**, 165–173 (2009).

88. K. Righter, R. Rowland, S. Yang, M. Humayun, Activity coefficients of siderophile elements in Fe-Si liquids at high pressure. *Geochem. Perspect. Lett.* **15**, 44–49 (2020).

89. R. Hultgren, P. D. Desai, D. T. Hawkins, M. Gleiser, K. K. Kelley, *Selected values of the thermodynamic properties of binary alloys* (1973).

90. L. J. Swartzendruber, B. Sundman, TheFe-Os(Iron-Osmium) System. *Bull. Alloy Phase Diagr.* **4**, 396–399 (1983).

91. JSPS-NCS, The Japan Society for the Promotion of Science and the nineteenth committee on steelmaking Part 2: Recommended values of activity and activity coefficients, and interaction parameters of elements in iron alloys. *Steelmaking Data Sourcebook* (1988), pp. 273–297.

92. U. Mann, D. J. Frost, D. C. Rubie, Evidence for high-pressure core-mantle differentiation from the metal-silicate partitioning of lithophile and weakly-siderophile elements. *Geochim. Cosmochim. Acta* **73**, 7360–7386 (2009).

93. K. Righter, L. Danielson, K. M. Pando, N. Marin, K. Nickodem, Origin of volatiles in Earth: Indigenous versus exogenous sources based on highly siderophile, volatile, siderophkle and light volatile elements. *Lunar and Planetary Science Conference* (2015), pp. 2–3.

94. A. Morbidelli, J. I. Lunine, D. P. O'Brien, S. N. Raymond, K. J. Walsh, Building terrestrial planets. *Ann. Rev. Earth Planet. Sci.* **40**, 251–275 (2012).

95. B. J. Wood, M. J. Walter, J. Wade, Accretion of the Earth and segregation of its core. *Nature* **441**, 825–833 (2006).

96. K. Tsuno, D. S. Grewal, R. Dasgupta, Core-mantle fractionation of carbon in Earth and Mars: The effects of sulfur. *Geochim. Cosmochim. Acta* **238**, 477–495 (2018).

97. A. Johansen, T. Ronnet, M. Bizzarro, M. Schiller, M. Lambrechts, Å. Nordlund, H. Lammer, A pebble accretion model for the formation of the terrestrial planets in the solar system. *Sci. Adv.* **7**, eabc0444 (2021).

98. A. Maller, M. Landeau, L. Allibert, S. Charnoz, Condition for metal fragmentation during Earth-forming collisions. *Phys. Earth Planet. In.* **352**, 107199 (2024).

99. M. Landeau, R. Deguen, D. Phillips, J. A. Neufeld, V. Lherm, S. B. Dalziel, Metal-silicate mixing by large Earth-forming impacts. *Earth Planet. Sci. Lett.* **564**, 116888 (2021).

100. W. F. McDonough, S. Sun, The composition of the Earth. *Chem. Geol.* **120**, 223–253 (1995).

101. H. Palme, H. S. C. O'Neill, Cosmochemical Estimates of Mantle Composition. in *Treatise on Geochemistry 2nd Edition* (Elsevier Ltd., ed. 2, 2014; http://dx.doi.org/10.1016/B978-0-08-095975-7.00201-1), pp. 1–39.

102. M. Fischer-Gödde, H. Becker, F. Wombacher, Rhodium, gold and other highly siderophile elements in orogenic peridotites and peridotite xenoliths. *Chem. Geol.* **280**, 365–383 (2011).

103. V. J. M. Salters, A. Stracke, Composition of the depleted mantle. *Geochem. Geophys. Geosyst.* **5**, Q05B07 (2004).

104. S. Aulbach, J. E. Mungall, D. G. Pearson, Distribution and processing of highly siderophile elements in cratonic mantle lithosphere. *Rev. Mineral. Geochem.* **81**, 239–304 (2016).

105. S. J. Barnes, J. E. Mungall, W. D. Maier, Platinum group elements in mantle melts and mantle samples. *Lithos* **232**, 395–417 (2015).

106. J. M. D. Day, R. J. Walker, J. M. Warren, 186Os–187Os and highly siderophile element abundance systematics of the mantle revealed by abyssal peridotites and Os-rich alloys. *Geochim. Cosmochim. Acta* **200**, 232–254 (2017).

107. E. Ito, K. Morooka, O. Ujike, T. Katsura, Reactions between molten iron and silicate melts at high pressure: Implications for the chemical evolution of Earth's core. *J. Geophys. Res.* **100**, 5901–5910 (1995).

108. C. K. Geßmann, D. C. Rubie, The effect of temperature on the partitioning of nickel, cobalt, manganese, chromium, and vanadium at 9 GPa and constraints on formation of the Earth's core. *Geochim. Cosmochim. Acta* **62**, 867–882 (1998).

109. A. Corgne, S. Keshav, B. J. Wood, W. F. McDonough, Y. Fei, Metal-silicate partitioning and constraints on core composition and oxygen fugacity during Earth accretion. *Geochim. Cosmochim. Acta* **72**, 574–589 (2008).

110. J. Siebert, A. Corgne, F. J. Ryerson, Systematics of metal-silicate partitioning for many siderophile elements applied to Earth's core formation. *Geochim. Cosmochim. Acta* **75**, 1451–1489 (2011).

111. K. Tsuno, D. J. Frost, D. C. Rubie, Simultaneous partitioning of silicon and oxygen into the Earth's core during early Earth differentiation. *Geophys. Res. Lett.* **40**, 66–71 (2013).

112. J. Badro, J. Aubert, K. Hirose, R. Nomura, I. Blanchard, S. Borensztajn, J. Siebert, Magnesium partitioning between earth's mantle and core and its potential to drive an early exsolution geodynamo. *Geophys. Res. Lett.* **45**, 13,240–13,248 (2018).

113. M. A. Bouhifd, A. P. Jephcoat, Convergence of Ni and Co metal-silicate partition coefficients in the deep magma-ocean and coupled silicon-oxygen solubility in iron melts at high pressures. *Earth Planet. Sci. Lett.* **307**, 341–348 (2011).

114. Z. Du, C. Jackson, N. Bennett, P. Driscoll, J. Deng, K. K. M. Lee, E. Greenberg, V. B. Prakapenka, Y. Fei, Insufficient energy from MgO exsolution to power early geodynamo. *Geophys. Res. Lett.* **44**, 11376–11381 (2017).

115. C. R. M. Jackson, N. R. Bennett, Z. Du, E. Cottrell, Y. Fei, Early episodes of high-pressure core formation preserved in plume mantle. *Nature* **553**, 491–495 (2018).
